# CARMN loss promotes VSMC-derived foam cell formation and atherosclerosis through transcriptional downregulation of autophagy

**DOI:** 10.1038/s41419-025-08157-z

**Published:** 2025-11-10

**Authors:** Tiegen Huang, Yao Tang, Quanli Su, Chen Su, Jiahao Wang, Lu Fu, Yixin Tang

**Affiliations:** 1 The First Affiliated Hospital, Department of Cardiology, Hengyang Medical School, University of South China, Hengyang, Hunan China; 2 The First Affiliated Hospital, Department of Cardiology, Harbin Medical University, Harbin, Heilongjiang China; 3 Hunan Provincial Key Laboratory of Multi-omics And Artificial Intelligence of Cardiovascular Diseases, University of South China, Hengyang, Hunan China

**Keywords:** Mechanisms of disease, Macroautophagy, Atherosclerosis

## Abstract

Vascular smooth muscle cells (VSMCs) are a significant source of foam cells in atherosclerosis, but the mechanism involved in the formation of VSMC-derived foam cells remains poorly understood. Although long noncoding RNAs (lncRNAs) are dysregulated in lipid metabolism disorder and atherosclerosis, little is known about their involvement in VSMC-derived foam cell formation. Silencing CARMN promoted lipid accumulation and VSMC-derived foam cell formation in high-fat diet-fed ApoE^−/−^ mice. Furthermore, CARMN knockdown reduced cholesterol efflux and promoted VSMC-derived foam cell formation by regulating autophagy. In exploring the mechanism by which CARMN regulates autophagy, our results demonstrated that CARMN knockdown reduced autophagy in VSMCs by modulating the AKT/ATG7 pathway through transcriptional suppression of the adjacent gene casein kinase 1 alpha 1 (CSNK1A1). In vivo, CARMN deficiency led to reduced autophagy in VSMCs and increased atherosclerotic lesions, characterized by increased lipid deposition and necrotic core. Our findings reveal that CARMN plays an essential role in the regulation of VSMC autophagy, which is crucial for the formation of VSMC-derived foam cells and the progression of atherosclerosis. These results provide new insights into the molecular mechanisms underlying VSMC autophagy and suggest that CARMN is a potential therapeutic target for atherosclerosis.

## Introduction

Atherosclerosis is a chronic inflammatory vascular disease characterized by endothelial dysfunction, lipid metabolism disorder, and lipid accumulation within the arterial wall, serving as the primary pathological basis for atherosclerotic cardiovascular diseases (ASCVD), such as coronary heart disease, cerebral infarction, and peripheral artery disease [1, 2]. Although antiplatelet and lipid-lowering therapies have successfully slowed atherosclerosis progression and reduced many complications, ASCVD remains the leading cause of death worldwide [3]. Foam cell formation caused by excessive cholesterol accumulation is the hallmark pathological feature of atherosclerotic lesions and was initially believed to originate from macrophages. However, recent findings have shown that at least 50% of foam cells in atherosclerotic lesions originate from vascular smooth muscle cells (VSMCs) [4–6]. Little is known about the mechanisms underlying VSMC-derived foam cell formation. Therefore, revealing these underlying mechanisms in atherosclerotic lesions may help ameliorate ASCVD.

Autophagy, a lysosome-dependent self-protective catabolic pathway in cells, plays a critical regulatory role in atherosclerosis [7]. Physiological autophagy exerts antiatherosclerotic effects by promoting cholesterol efflux, attenuating inflammatory responses, or inhibiting apoptosis. Conversely, suppressed or overactivated autophagy contributes to proatherosclerotic impacts through impaired cholesterol efflux, exacerbated inflammation, or enhanced apoptosis [8–10]. Lipophagy is a type of selective autophagy that is thought to play a role in lipid metabolism by transporting intracellular lipid droplets (LDs) to lysosomes for degradation [11]. Defective lipophagy has been linked to lipid-related diseases such as fatty liver and atherosclerosis [12]. Inhibition of VSMC autophagy promotes atherosclerosis progression by facilitating VSMC-derived foam cell formation [13]. Promoting VSMC autophagy represents a potentially promising therapeutic strategy for atherosclerosis. Therefore, identifying key molecules and mechanisms that regulate autophagy in VSMCs is essential for developing effective therapies.

Long noncoding RNAs (lncRNAs) are RNA transcripts longer than 200 nucleotides and lack protein-coding potential. LncRNAs were initially thought to represent transcriptional “noise” and considered biologically nonfunctional due to their inability to encode proteins [14]. However, they play important roles in atherosclerosis by regulating genetic programming and cellular function [15]. Therefore, identifying key lncRNAs associated with atherosclerosis and exploring their mechanisms may provide valuable insights into the pathogenesis and progression of this disease.

To explore the mechanisms underlying the progression of atherosclerosis, we performed bioinformatics analysis on the GSE97210 dataset, which included three human normal arterial intimal tissues and three human atherosclerotic plaque tissues. We further used C57BL/6J mice fed a normal chow diet for 8 weeks as the control group and ApoE^−/−^ mice fed a high-fat diet (HFD) for 8 weeks as the atherosclerosis model group. Our analysis revealed significant differences in gene expression between normal arterial intima and atherosclerotic plaque tissues. This study focused on atherosclerosis-associated lncRNAs and identified CARMN as a highly expressed lncRNA in atherosclerotic plaques. Recent studies have demonstrated that CARMN knockdown promotes VSMC dedifferentiation by downregulating miR-145 and miR-143 expression, ultimately accelerating atherosclerosis progression [16]. However, in vitro experiments revealed that exogenous supplementation with miR-145 and miR-143 did not fully reverse the proatherogenic effects induced by CARMN silencing, suggesting that CARMN knockdown may promote atherosclerosis progression through additional mechanisms [17]. Therefore, investigating the novel mechanism of CARMN in atherosclerosis may provide important insights into the pathogenesis and progression of atherosclerosis. Furthermore, studying CARMN could clarify the complex interaction between VSMC autophagy and atherosclerosis, suggesting novel targets for therapeutic intervention.

## Results

### Silencing CARMN promotes atherosclerosis and VSMC-derived foam cell formation

**Fig. 1:**
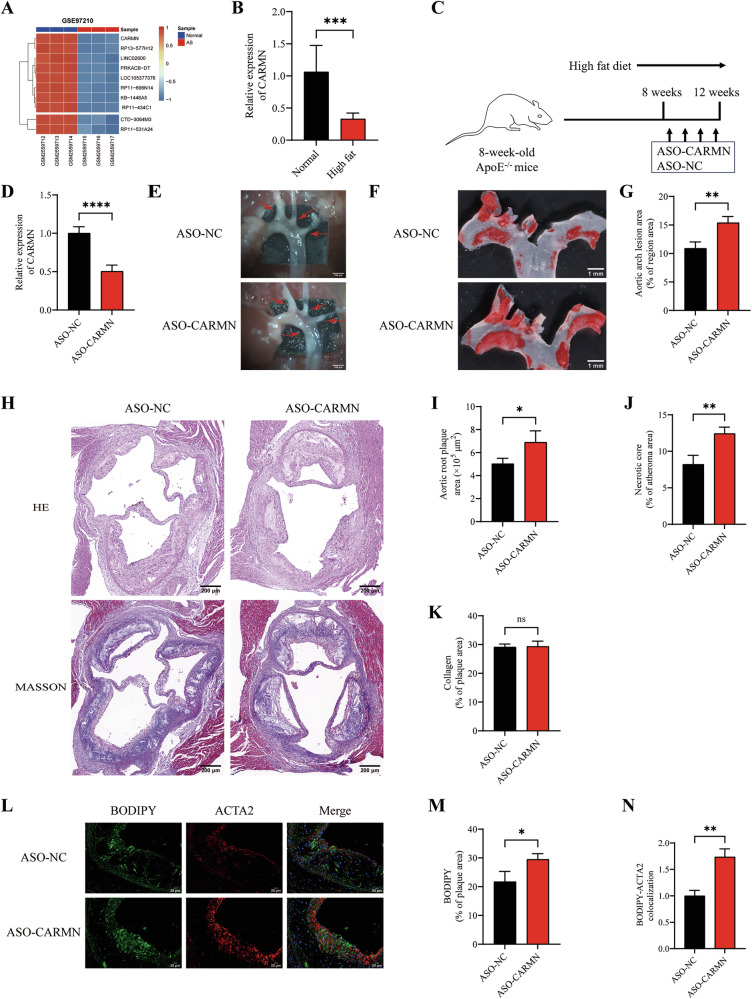
Silencing CARMN promotes atherosclerosis and VSMC-derived foam cell formation. **A** The heatmap illustrates the expression levels of the top 10 downregulated lncRNAs in the atherosclerosis group compared to the normal group. **B** qRT-PCR analysis of CARMN in aortic tissues extracted from normal control mice and atherosclerotic mice (*n* = 6). **C** Schematic representation of the atherosclerosis model and grouping. ApoE^−/−^ mice at 8 weeks of age were fed a HFD for 12 weeks (8 weeks of induction followed by 4 weeks of maintenance), and tail vein was injected with ASO-CARMN (10 nmol per mouse) or ASO-NC (10 nmol per mouse) twice weekly during the last 4 weeks. The mice were then euthanized for collection of aortic tissue and follow-up examinations (*n* = 6). **D** The knockdown efficiency of CARMN was determined by qRT-PCR analysis of RNA extracted from the aortic tissues of mice in ASO-NC and ASO-CARMN groups (*n* = 3). **E** The plaques (red arrows) in the aortic arch of ApoE^−/−^ mice under a stereoscopic microscope (*n* = 6). Scale bars, 100 µm. **F** En face ORO staining of the aortic arch regions in ASO-NC and ASO-CARMN groups (*n* = 6). Scale bars, 1 mm. **G** Quantification of the percentage of en face ORO-positive area/aortic arch area (*n* = 6). **H** Representative images of aortic root sections stained with HE and Masson staining, respectively. Scale bars, 200 µm. **I** Quantitative data of the atherosclerotic plaque area in the aortic roots (*n* = 6). **J**, **K** Quantification of necrotic core area and collagen-containing area as a percentage of the total plaque area (*n* = 6). **L** Representative images of immunofluorescent staining showing BODIPY-ACTA2 colocalization in aortic root sections from ASO-NC and ASO-CARMN groups. Nuclei were stained with DAPI (blue). Scale bars, 20 µm. **M**, **N** The percentages of BODIPY-positive area and BODIPY-ACTA2 colocalization in the plaque area (*n* = 3). Data are presented as mean ± SD. Statistical analysis was performed using the two-tailed Student’s t-test for two-group comparisons. ns, *P* > 0.05; *, *P* < 0.05; **, *P* < 0.01; ***, *P* < 0.001; ****, *P* < 0.0001.

To explore the mechanisms underlying the progression of atherosclerosis, we performed bioinformatics analysis on the GSE97210 dataset, which included 3 human normal arterial intimal tissues and 3 human atherosclerotic plaque tissues. Principal component analysis (PCA) revealed evident segregation between normal arterial intimal tissues and atherosclerotic plaque tissues, reflecting substantial differences in gene expression profiles (Fig. S1A). The volcano plot shows the distribution of differentially expressed lncRNAs in the two groups (Fig. S1B). By analyzing the differentially expressed lncRNAs in the atherosclerosis group compared with the normal group, we identified the lncRNA CARMN as one of the top 10 downregulated lncRNAs in atherosclerotic plaques (Figs. 1A and S1C). We used the Basic Local Alignment Search Tool (BLAST) to assess the conservation of the top 10 downregulated lncRNAs and found that CARMN is a highly conserved lncRNA expressed in both humans and mice. The human CARMN transcript (ENST00000519898.7) and the mouse CARMN transcript (ENSMUST00000182244.9) presented high sequence similarity and conserved synteny (72% homology, Fig. S1D, E). CARMN is annotated as a lncRNA in both the NCBI and Ensembl databases, and its low coding potential has been demonstrated in a previous study [18]. Bioinformatics analysis revealed that CARMN was among the top 10 downregulated lncRNAs in atherosclerotic plaques, which was further validated by quantitative real-time polymerase chain reaction (qRT-PCR) in the aortic tissues of normal control mice and atherosclerotic mice (Fig. 1B).

To further investigate the function of CARMN in atherosclerosis models, we used antisense oligonucleotides (ASOs) to knock it down. We first established an atherosclerotic plaque model in 8-week-old ApoE^−/−^ mice by feeding them a HFD for 12 weeks (8 weeks of induction followed by 4 weeks of maintenance). During the last 4 weeks, ASO-CARMN (10 nmol per mouse) or ASO-NC (10 nmol per mouse) was administered via tail vein injection twice weekly. The mice were euthanized for collection of aortic tissue and follow-up examinations (Fig. 1C). The efficiency of CARMN knockdown in aortic tissue was confirmed by qRT-PCR (Fig. 1D). We found that CARMN knockdown increased the number and size of atherosclerotic lesions in the aortic arch regions (Fig. 1E). We also observed a significant increase in lipid deposition in ASO-CARMN-transfected mice compared with the ASO-NC group, as shown by oil red O (ORO) staining of the aortic arch (Fig. 1F, G). In addition, hematoxylin and eosin (HE) and Masson’s trichrome (Masson) staining revealed that CARMN knockdown resulted in a higher arterial plaque burden, with a significant increase in plaque size and necrotic core area in the cross-section of the aortic root (Fig. 1H–J). However, no significant differences in the collagen content were noted between the two groups (Fig. 1K). These results suggest that CARMN knockdown exerts a proatherosclerotic effect by increasing lipid deposition and necrotic core.

Recent studies in human atherosclerotic plaques have shown that at least 50% of foam cells are derived from VSMCs [4, 5]. Other studies have shown that CARMN is specifically expressed in VSMCs and that its expression progressively decreases with the progression of atherosclerosis [16]. To investigate whether CARMN regulates VSMC-derived foam cell formation, we labeled lipids with BODIPY (BODIPY staining is an indicator of neutral fat deposition) and labeled VSMCs in plaques with ACTA2 (ACTA2 is a specific marker for VSMCs) (Fig. 1L). The results revealed that lipid deposition and BODIPY-ACTA2 colocalization were significantly increased in the ASO-CARMN-transfected group compared to the ASO-NC-transfected group, suggesting that CARMN knockdown increased the number of VSMC-derived foam cells (Fig. 1M, N).

### CARMN knockdown promotes VSMC-derived foam cell formation by inhibiting cholesterol efflux

**Fig. 2:**
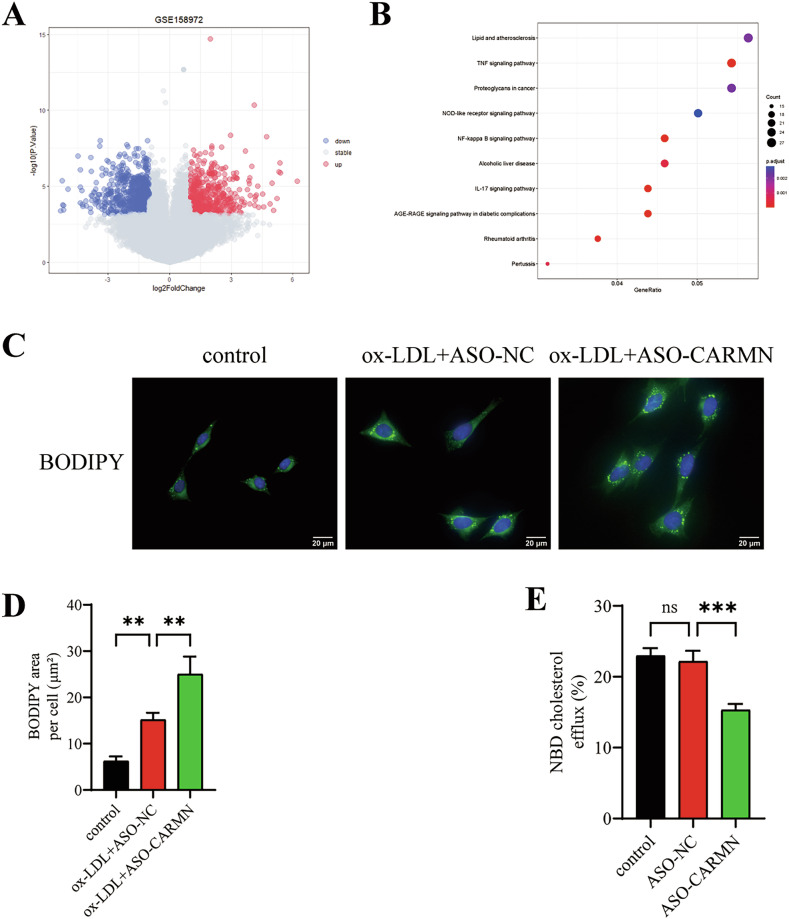
CARMN knockdown promotes VSMC-derived foam cell formation by inhibiting cholesterol efflux. **A** Volcano plot showing the DEGs in VSMCs with CARMN knockdown compared with control. Red and blue dots represent significantly upregulated and downregulated genes with |log_2_FC | > 1 and *P* < 0.01 as the threshold parameters. The gray dots indicate non-differentially expressed genes. **B** The KEGG enrichment analysis was conducted to identify the enriched pathways of the DEGs. **C** Representative images of BODIPY staining in VSMCs from the vehicle control, ox-LDL plus ASO-NC, and ox-LDL plus ASO-CARMN groups. Nuclei were stained with DAPI (blue). Scale bars, 20 µm. **D** The average BODIPY-positive area per cell was quantified (*n* = 3). **E** HDL-mediated cholesterol efflux (%) was measured in the indicated groups (*n* = 3). Data are presented as mean ± SD. Statistical analysis was performed using one-way ANOVA for multiple-group comparisons. ns, *P* > 0.05; **P < 0.01; ***P < 0.001.

Given that lipid deposition in VSMCs was increased upon CARMN knockdown in vivo, we investigated whether a similar change occurred in vitro. We screened the differentially expressed genes (DEGs) in human-derived control VSMCs and CARMN-knockdown VSMCs using the GSE158972 dataset. The volcano plot shows the distribution of DEGs in the two groups (Fig. 2A). Kyoto Encyclopedia of Genes and Genomes (KEGG) enrichment analysis of the resulting DEGs revealed significant enrichment in the “lipid-atherosclerosis” pathway (Fig. 2B), suggesting that CARMN modulates lipid metabolism in vitro.

To investigate whether CARMN is involved in regulating VSMC-derived foam cell formation in vitro, we treated VSMCs with vehicle control, ox-LDL plus ASO-NC, or ox-LDL plus ASO-CARMN. We visualized lipids in VSMCs using BODIPY staining and quantified the positive area. We observed a significant increase in intracellular lipid deposition in the ASO-CARMN-transfected group compared with the ASO-NC-transfected group (Fig. 2C, D), suggesting that knockdown of CARMN promotes VSMC-derived foam cell formation.

Decreased cholesterol efflux is the leading cause of intracellular lipid deposition [19, 20]. To explore whether CARMN regulates cholesterol efflux, we stimulated 22-(N-[7-nitrobenz-2-oxa-1,3-diazol-4-yl] amino)-23,24-bisnor-5-cholen-3β-ol (NBD-cholesterol)-loaded VSMCs with vehicle control, ASO-NC, or ASO-CARMN, and the efflux of the labeled cholesterol to high-density lipoprotein (HDL) was analyzed. We found that CARMN knockdown significantly inhibited cholesterol efflux (Fig. 2E). These results indicate that CARMN knockdown promotes VSMC-derived foam cell formation by inhibiting cholesterol efflux.

### Knockdown of CARMN transcriptionally downregulates autophagy in VSMCs

Differences in cholesterol efflux are typically attributed to alterations in the expression of ATP-binding cassette transporters. However, our quantification of ATP binding cassette subfamily A member 1 (ABCA1) and ABCG1 (ATP binding cassette subfamily G member 1) protein expression revealed no significant differences among the groups, suggesting that CARMN regulates cholesterol efflux through other mechanisms (Fig. S2A, B).

**Fig. 3:**
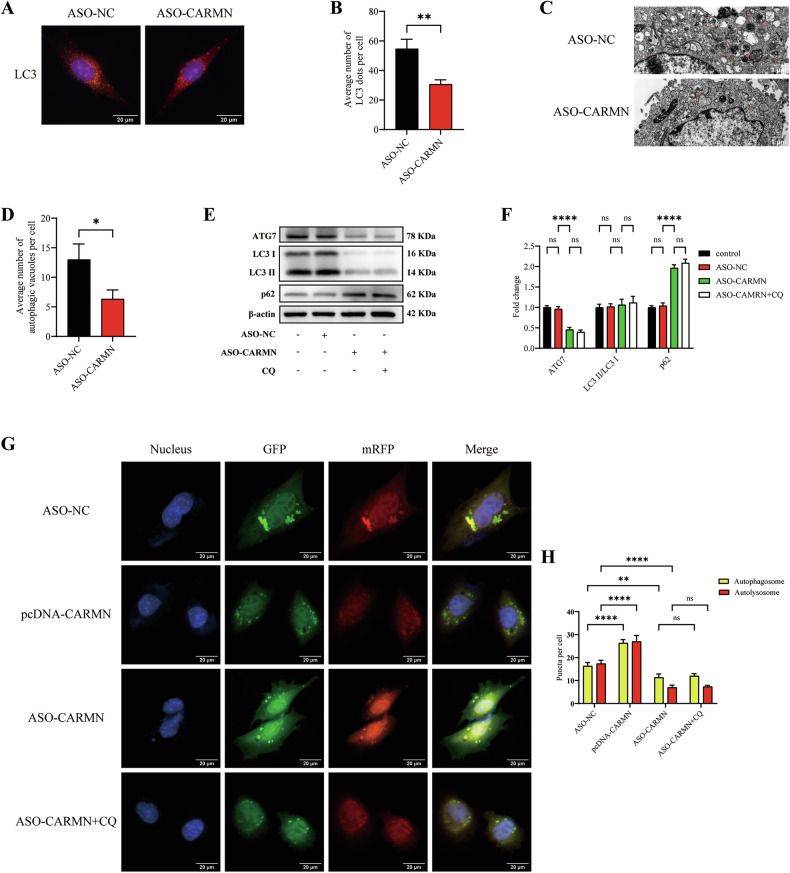
Knockdown of CARMN transcriptionally downregulates autophagy in VSMCs. **A** Representative images of LC3 staining in VSMCs transfected with ASO-NC or ASO-CARMN. Nuclei were stained with DAPI (blue). Scale bars, 20 µm. **B** The average number of LC3 dots per cell was quantified (*n* = 3). **C** Representative images of autophagic vacuoles (red arrows) observed by transmission electron microscopy in VSMCs transfected with ASO-NC or ASO-CARMN. Scale bars, 1 µm. **D** Quantification of autophagic vacuoles was performed on 15 randomly selected cells (five cells per experiment) across three independent replicates. **E** Western blotting analysis of the expression of LC3, p62, and ATG7 in VSMCs incubated with CQ (50 µM) for 12 h before treatment with ASO-NC or ASO-CARMN. **F** Quantification of LC3 II/LC3 I, p62 and ATG7 protein expression (*n* = 3). **G** VSMCs were transiently transfected with ad-mRFP-GFP-LC3 for 24 h and subsequently transfected with ASO-NC, pcDNA-CARMN, ASO-CARMN, or ASO-CARMN plus CQ (50 µM, 12 h). Scale bars, 20 μm. **H** Quantitative data on the number of autophagosomes (yellow puncta) and autolysosomes (red puncta) (*n* = 3). Data are presented as mean ± SD. Statistical analysis was performed using either the two-tailed Student’s t-test for two-group comparisons or one-way ANOVA for multiple-group comparisons. ns, *P* > 0.05; *, *P* < 0.05; **, *P* < 0.01; ****, *P* < 0.0001.

Autophagy, a lysosome-dependent self-protective catabolic pathway in cells, plays an important role in atherosclerosis by affecting cholesterol efflux [7]. To investigate whether CARMN regulates autophagy, we first stained microtubule-associated protein 1 light chain 3 (LC3) in VSMCs transfected with ASO-NC or ASO-CARMN. Compared with ASO-NC, ASO-CARMN significantly decreased the number of autophagic structures (LC3 dots), suggesting that CARMN knockdown affected autophagic activity (Fig. 3A, B). As expected, the number of autophagic vacuoles was decreased by ASO-CARMN (Fig. 3C, D). Autophagy is a dynamic process in which the formation of autophagosomes and their degradation by lysosomes occur simultaneously. The observation of LC3 dots using immunofluorescence staining alone cannot be used to determine whether autophagy is promoted or inhibited; it is often necessary to combine this method with autophagy tools for comprehensive analysis and judgment. Chloroquine (CQ) inhibits late autophagy [21], so we further analyzed the effect of CARMN in combination with CQ on autophagic activity. LC3 (both the LC3 I and LC3 II forms) and ATG7 expression was significantly decreased, whereas the expression of sequestosome 1 (p62) was markedly increased by ASO-CARMN. The LC3 II/LC3 I ratio remained unchanged, suggesting that ASO-CARMN reduces overall LC3 expression rather than specifically affecting lipidation. Interestingly, CQ had no obvious effect on the protein levels of LC3, p62, or ATG7 after treatment with ASO-CARMN (Fig. 3E, F). Conversely, overexpression of CARMN significantly increased the expression of LC3 and ATG7 while markedly decreasing the level of p62. The LC3 II/LC3 I ratio remained unchanged, further suggesting that CARMN primarily regulates the overall expression of LC3 rather than specifically affecting LC3 lipidation. After CQ treatment, LC3 (particularly LC3 II) and p62 levels were further elevated, with a significant increase in the LC3 II/LC3 I ratio, indicating that LC3 II accumulation was due to blocked autophagic degradation. Notably, ATG7 expression remained high in both the pcDNA-CARMN group and the pcDNA-CARMN combined with CQ group (Fig. S2C, D). Furthermore, we verified these effects using the autophagy flux reporter mRFP-GFP tandem fluorescent-tagged LC3 adenovirus (ad-mRFP-GFP-LC3). mRFP^+^ GFP^+^ LC3 puncta (yellow puncta) indicate autophagosomes, whereas mRFP^+^ GFP^−^ puncta (red puncta) represent autolysosomes. We found that both yellow and red puncta were significantly decreased by ASO-CARMN and increased by pcDNA-CARMN in mRFP-GFP-LC3 adenovirus-infected VSMCs. Consistent with the western blot results, CQ had no obvious effect on the numbers of yellow or red puncta after treatment with ASO-CARMN (Fig. 3G, H). Collectively, these results indicate that CARMN knockdown does not inhibit autophagic flux at the degradation stage but instead induces the transcriptional downregulation of autophagy-related genes, such as LC3 and ATG7, thereby resulting in reduced autophagy initiation.

### CARMN knockdown reduces cholesterol efflux and promotes VSMC-derived foam cell formation by regulating autophagy

**Fig. 4:**
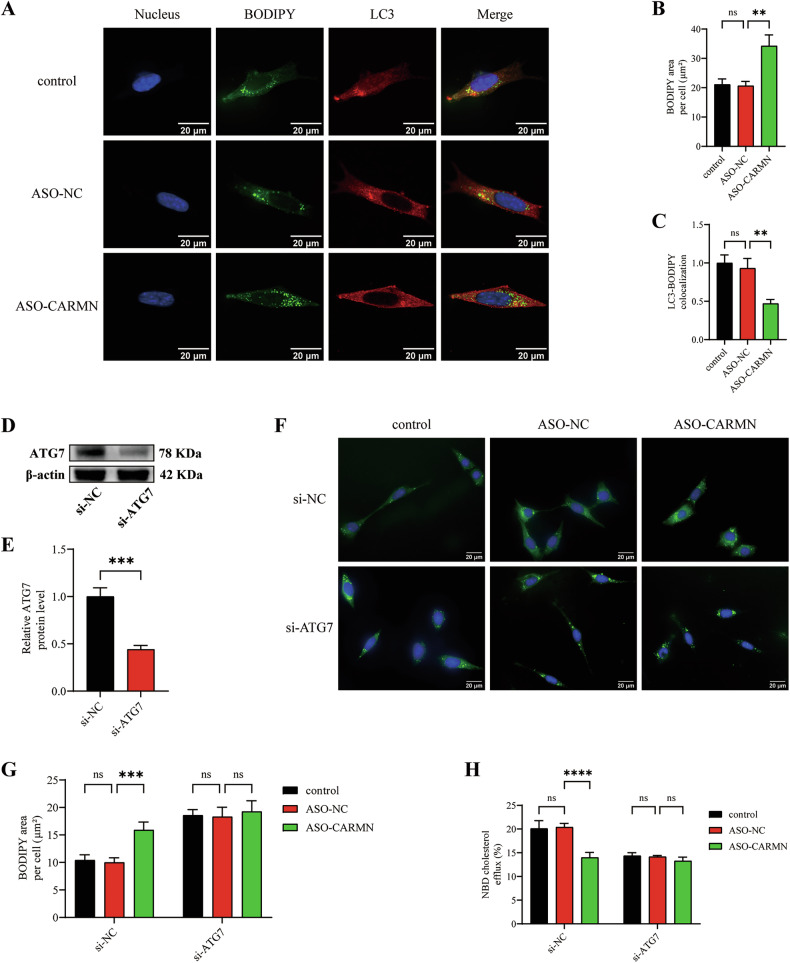
CARMN knockdown reduces cholesterol efflux and promotes VSMC-derived foam cell formation by regulating autophagy. **A** Representative images of immunofluorescent staining showing LC3-BODIPY colocalization in VSMCs pretreated with ox-LDL (50 μg/mL) and then treated with vehicle control, ASO-NC, or ASO-CARMN. Nuclei were stained with DAPI (blue). Scale bars, 20 µm. **B** The average BODIPY-positive area per cell was quantified (*n* = 3). **C** The percentages of LC3-BODIPY colocalization in VSMCs (*n* = 3). **D** Western blotting analysis of the expression of ATG7 in VSMCs transfected with a control siRNA (si-NC) or an ATG7 siRNA (si-ATG7). **E** Quantification of ATG7 protein expression (*n* = 3). **F** Representative images of BODIPY staining in VSMCs from vehicle control, ASO-NC, or ASO-CARMN groups treated with either si-NC or si-ATG7. Nuclei were stained with DAPI (blue). Scale bars, 20 µm. **G** The average BODIPY-positive area per cell was quantified (*n* = 3). **H** HDL-mediated cholesterol efflux (%) was measured in the indicated groups (*n* = 3). Data are presented as mean ± SD. Statistical analysis was performed using either the two-tailed Student’s t-test for two-group comparisons or one-way ANOVA for multiple-group comparisons. ns, *P* > 0.05; **, *P* < 0.01; ***, *P* < 0.001; ****, *P* < 0.0001.

To investigate whether CARMN affects lipid metabolism by regulating autophagy, we first detected a direct interaction between LDs and autophagic structures by immunofluorescence staining. We used an LC3 antibody to label autophagic structures and BODIPY to label LDs in VSMCs. We found that CARMN knockdown increased LDs but decreased the colocalization of LC3-BODIPY, suggesting that CARMN knockdown increased intracellular lipid deposition by influencing autophagy (Fig. 4A–C).

To further verify whether decreased autophagy could explain the decrease in cholesterol efflux and increase in lipid deposition caused by knockdown of CARMN, we transfected VSMCs with siRNA to knock down autophagy-related 7 (ATG7), which is a key protein in the autophagic process (Fig. 4D, E). We found that increased lipid deposition and decreased cholesterol efflux were particularly effective in the ASO-CARMN-transfected group than in the ASO-NC-transfected group of si-NC-transfected VSMCs. In contrast, in si-ATG7-transfected VSMCs, no significant differences in lipid deposition or cholesterol efflux were noted between the groups (Fig. 4F–H). Collectively, these results suggest that the knockdown of CARMN in VSMCs reduces cholesterol efflux and promotes foam cell formation by influencing autophagy.

### CARMN knockdown transcriptionally downregulates autophagy via the AKT/ATG7 pathway

**Fig. 5:**
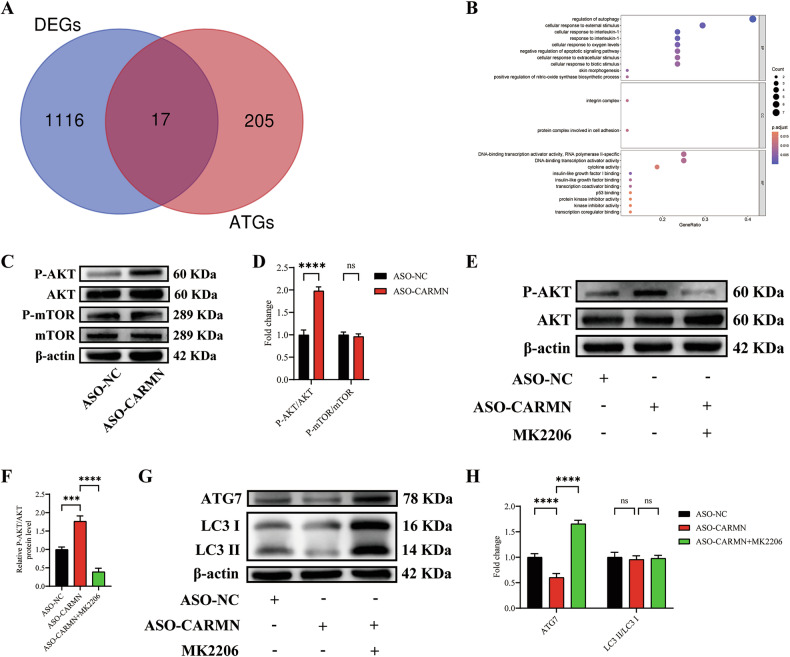
CARMN knockdown transcriptionally downregulates autophagy via the AKT/ATG7 pathway. **A** The Venn plot of the differentially expressed ATGs. **B** The GO enrichment analysis was performed to identify the biological process (BP), cellular component (CC), and molecular function (MF) of the differentially expressed ATGs. **C** Western blotting analysis of total and phosphorylated forms of AKT and mTOR in VSMCs treated with ASO-NC or ASO-CARMN. **D** Quantification of total and phosphorylated levels of AKT and mTOR (*n* = 3). **E** Western blotting analysis of total and phosphorylated forms of AKT in VSMCs incubated with MK2206 (5 µM) for 6 h before treatment with ASO-NC or ASO-CARMN. **F** Quantification of total and phosphorylated levels of AKT (*n* = 3). **G** Western blotting analysis of LC3 and ATG7 in VSMCs incubated with MK2206 (5 µM) for 6 h before treatment with ASO-NC or ASO-CARMN. **H** Quantification of LC3 II/LC3 I and ATG7 protein expression (*n* = 3). Data are presented as mean ± SD. Statistical analysis was performed using one-way ANOVA for multiple-group comparisons. ns, *P* > 0.05; ***, *P* < 0.001; ****, *P* < 0.0001.

To further investigate the mechanism by which CARMN regulates autophagy, we first intersected the DEGs in the GSE158972 dataset with 222 autophagy-related genes (ATGs) in the human autophagy database and identified 17 differentially expressed ATGs (Fig. 5A). Gene Ontology (GO) enrichment analyses demonstrated that the differentially expressed ATGs were enriched in autophagy (Fig. 5B). The “autophagy-animal” category of KEGG signaling pathways was visualized, and six autophagy-related genes (HIF1α, Raptor, DAPK, ATG9L, ATG10, and FLIP) were dispersedly distributed in the nucleation process of autophagic vacuoles, which verified that CARMN knockdown reduced the initiation stage of autophagy (Fig. S3A).

The initiation phase of autophagy is regulated by two main mechanisms: 1) direct regulation of the initial step of vesicle nucleation and 2) regulation of signaling pathways upstream of autophagy, such as the AMP-activated protein kinase (AMPK) or the AKT-mTOR signaling pathway [22, 23]. Increasing evidence suggests that the AKT-mTOR signaling pathway plays a key role in autophagy [24, 25]. Therefore, we examined whether CARMN regulates autophagy through the classical AKT-mTOR signaling pathway. In our study, we found that the phosphorylation level of AKT was significantly increased by ASO-CARMN, whereas mTOR phosphorylation levels and total AKT and mTOR protein expression levels did not change significantly (Fig. 5C, D). We also found that ASO-CARMN-induced increases in AKT phosphorylation were suppressed by MK-2206 2 HCL (MK2206, an AKT inhibitor) (Fig. 5E, F). These data suggest that CARMN does not regulate autophagy through the classical AKT-mTOR signaling pathway.

Our previous experimental results indicated that ATG7 plays an important role in autophagy and lipid metabolism, so we further investigated whether CARMN regulates autophagy by affecting the expression of the AKT/ATG7 pathway. We found that LC3 (both the LC3 I and LC3 II forms) and ATG7 expression levels, which were suppressed by ASO-CARMN, were significantly increased by MK2206, whereas the LC3 II/LC3 I ratio remained unchanged (Fig. 5G, H). However, the suppressed expression levels of LC3 and ATG7 could not be restored by rapamycin (Rapa, an mTOR inhibitor and autophagy agonist), further indicating that CARMN regulates autophagy in an mTOR-independent manner (Fig. S3B, C). Thus, these results indicate that CARMN knockdown transcriptionally downregulates autophagy via the AKT/ATG7 pathway.

To confirm the involvement of AKT in regulating cholesterol efflux and ultimately lipid deposition, we used MK2206 to inhibit AKT activity. We found that MK2206 significantly decreased lipid deposition and increased cholesterol efflux in VSMCs, suggesting that MK2206 can reverse the ability of ASO-CARMN to promote lipid deposition and inhibit cholesterol efflux (Fig. S3D, E). Therefore, we conclude that CARMN knockdown transcriptionally downregulates autophagy via the AKT/ATG7 pathway, which inhibits cholesterol efflux and promotes lipid deposition.

### CARMN knockdown inhibits CSNK1A1 transcription

In most cases, lncRNAs exert their functions by regulating the expression of target mRNAs at the transcriptional or posttranscriptional level [26]. To further elucidate the potential mechanism of CARMN, we first considered the distribution of CARMN in VSMCs. A recent study confirmed that CARMN is located mainly in the nucleus and could be involved in the development of atherosclerosis by regulating the expression of adjacent genes [27]. We first identified adjacent genes within 100 kb upstream and downstream of CARMN using the Ensembl database (https://asia.ensembl.org/index.html) and then calculated the interaction probabilities between CARMN and these adjacent genes (http://pridb.gdcb.iastate.edu/RPISeq/). We found that CARMN potentially binds to CSNK1A1 (RF: 0.65, RF scores > 0.5 were considered “positive”) (Fig. S4A, B).

**Fig. 6:**
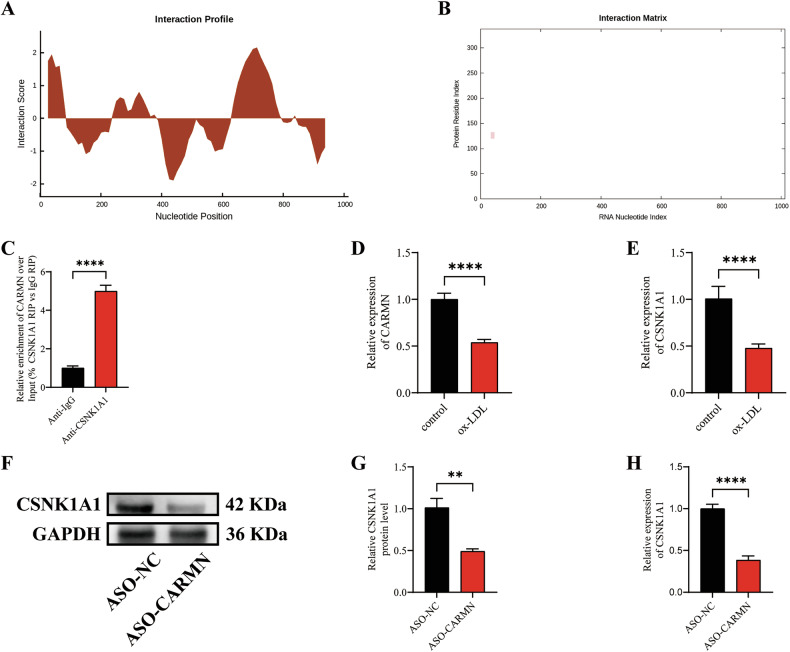
CARMN knockdown inhibits CSNK1A1 transcription. **A** The interaction profile, indicating the protein binding affinity (Y-pathway) across the CARMN RNA sequence (X-pathway), reveals the most probable protein-binding region. **B** The figure shows the location of a significant interaction between CSNK1A1 protein and CARMN RNA, highlighted in red at the corresponding coordinates on the X and Y axes. **C** RIP assays were performed to validate CARMN binding to CSNK1A1 in VSMCs (*n* = 3). **D**, **E** qRT-PCR analysis of CARMN and CSNK1A1 mRNA levels in VSMCs treated with vehicle control or ox-LDL (50 μg/mL) for 24 h (*n* = 3). **F** Western blotting analysis of the expression of CSNK1A1 in VSMCs treated with ASO-NC or ASO-CARMN. **G** Quantification of CSNK1A1 protein expression (*n* = 3). **H** qRT-PCR analysis of CSNK1A1 mRNA levels in VSMCs treated with ASO-NC or ASO-CARMN (*n* = 3). Data are presented as mean ± SD. Statistical analysis was performed using the two-tailed Student’s t-test for two-group comparisons. **P < 0.01; ****P < 0.0001.

To further identify the potential binding regions between CARMN and CSNK1A1, we used the CatRAPID online algorithm (http://service.tartaglialab.com/page/catrapid_group), which rapidly predicts RNA-protein interaction size and binding regions [28]. CatRAPID analysis revealed that the 26 bp-77 bp region of CARMN was most likely to bind to CSNK1A1 (Fig. 6A, B). We subsequently performed RNA Immunoprecipitation (RIP) experiments in VSMCs, and the results confirmed that CARMN binds directly to CSNK1A1 (Fig. 6C).

To confirm whether CARMN plays a role in atherosclerosis by regulating CSNK1A1 expression, we stimulated VSMCs with ox-LDL and detected the expression of CARMN and CSNK1A1 using qRT-PCR. We found that the expression of both CARMN and CSNK1A1 was reduced by ox-LDL stimulation (Fig. 6D, E). In addition, we found that CARMN knockdown in VSMCs significantly reduced CSNK1A1 expression at the protein and mRNA levels (Fig. 6F–H). Taken together, these results indicate that CARMN knockdown significantly inhibits the transcription level of the adjacent gene CSNK1A1.

### CSNK1A1 knockdown transcriptionally downregulates autophagy via the AKT/ATG7 pathway

**Fig. 7:**
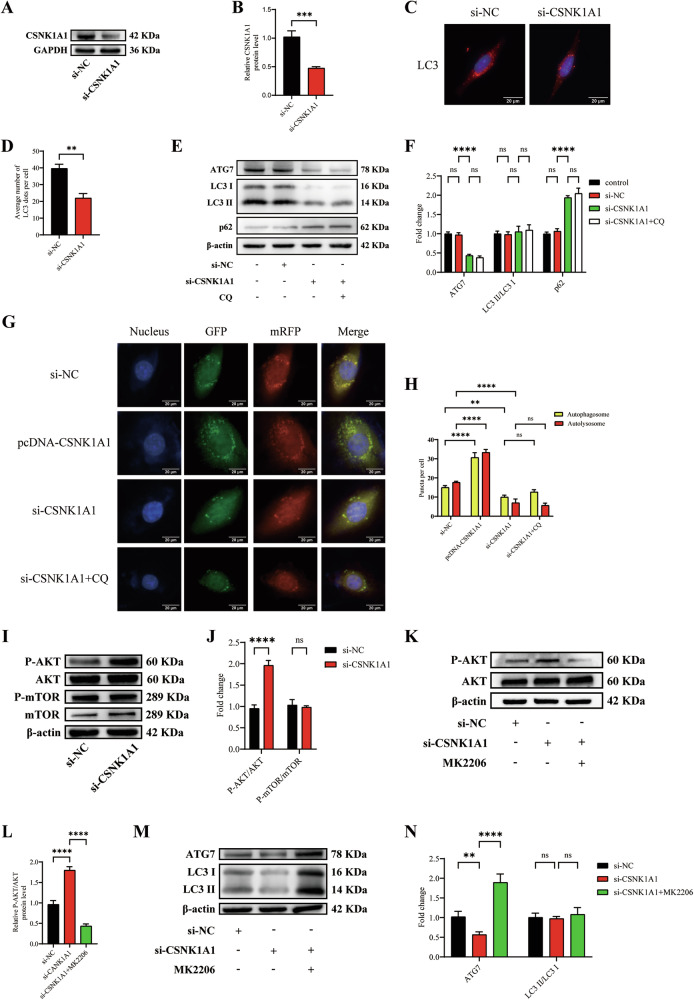
CSNK1A1 knockdown transcriptionally downregulates autophagy via the AKT/ATG7 pathway. **A** Western blotting analysis of the expression of CSNK1A1 in VSMCs transfected with a control siRNA (si-NC) or a CSNK1A1 siRNA (si-CSNK1A1). **B** Quantification of CSNK1A1 protein expression (*n* = 3). **C** Representative images of LC3 staining in VSMCs transfected with si-NC or si-CSNK1A1. Nuclei were stained with DAPI (blue). Scale bars, 20 µm. **D** The average number of LC3 dots per cell was quantified (*n* = 3). **E** Western blotting analysis of the expression of LC3, p62, and ATG7 in VSMCs incubated with CQ (50 µM) for 12 h before treatment with si-NC or si-CSNK1A1. **F** Quantification of LC3 II/LC3 I, p62 and ATG7 protein expression (*n* = 3). **G** VSMCs were transiently transfected with ad-mRFP-GFP-LC3 for 24 h and subsequently transfected with si-NC, pcDNA-CSNK1A1, si-CSNK1A1, or si-CSNK1A1 plus CQ (50 µM, 12 h). Scale bars, 20 μm. **H** Quantitative data on the number of autophagosomes (yellow puncta) and autolysosomes (red puncta) (*n* = 3). **I** Western blotting analysis of total and phosphorylated forms of AKT and mTOR in VSMCs treated with si-NC or si-CSNK1A1. **J** Quantification of total and phosphorylated levels of AKT and mTOR (*n* = 3). **K** Western blotting analysis of total and phosphorylated forms of AKT in VSMCs incubated with MK2206 (5 µM) for 6 h before treatment with si-NC or si-CSNK1A1. **L** Quantification of total and phosphorylated levels of AKT (*n* = 3). **M** Western blotting analysis of LC3 and ATG7 in VSMCs incubated with MK2206 (5 µM) for 6 h before treatment with si-NC or si-CSNK1A1. **N** Quantification of LC3 II/LC3 I and ATG7 protein expression (*n* = 3). Data are presented as mean ± SD. Statistical analysis was performed using either the two-tailed Student’s t-test for two-group comparisons or one-way ANOVA for multiple-group comparisons. ns, *P* > 0.05; **, *P* < 0.01; ***, *P* < 0.001; ****, *P* < 0.0001.

Previous studies have shown that CSNK1A1 inhibits the growth of non-small cell lung cancer by inducing autophagy through the AKT/FOXO3a/ATG7 pathway [29]. However, it remains unclear whether CSNK1A1 modulates atherosclerosis progression by regulating autophagy. To explore the potential mechanism by which CSNK1A1 contributes to atherosclerosis, we first transfected VSMCs with siRNA to knock down CSNK1A1 (Fig. 7A, B). Compared with the si-NC-transfected group, the si-CSNK1A1-transfected group presented significantly fewer LC3 dots, suggesting that the knockdown of CSNK1A1 affected autophagic activity (Fig. 7C, D). LC3 (both the LC3 I and LC3 II forms) and ATG7 expression was significantly decreased, whereas p62 expression was markedly increased by si-CSNK1A1. The LC3 II/LC3 I ratio remained unchanged, suggesting that si-CSNK1A1 reduces overall LC3 expression rather than specifically affecting lipidation. Interestingly, CQ had no obvious effect on LC3, p62, or ATG7 protein levels after treatment with si-CSNK1A1 (Fig. 7E, F). We also found that yellow and red puncta were significantly decreased by si-CSNK1A1 and increased by pcDNA-CSNK1A1 in mRFP-GFP-LC3 adenovirus-infected VSMCs. CQ had no obvious effect on the yellow or red puncta after treatment with si-CSNK1A1 (Fig. 7G, H). These results indicate that the knockdown of CSNK1A1 does not inhibit autophagic flux at the degradation stage but instead induces the transcriptional downregulation of autophagy-related genes, such as LC3 and ATG7, thereby resulting in reduced autophagy initiation.

To investigate whether CARMN knockdown regulates autophagy in VSMCs by regulating CSNK1A1 transcription, we transfected VSMCs with ASO-CARMN or ASO-CARMN plus pcDNA-CSNK1A1. We found that the ASO-CARMN-induced increase in AKT phosphorylation levels was significantly suppressed by pcDNA-CSNK1A1 (Fig. S5A, B). Furthermore, the reduction in LC3 and ATG7 expression caused by ASO-CARMN was reversed by pcDNA-CSNK1A1, while the LC3 II/LC3 I ratio remained unchanged (Fig. S5C, D). These results indicate CARMN knockdown regulates autophagy in VSMCs by regulating the transcription of CSNK1A1.

To further investigate the mechanism by which CSNK1A1 regulates autophagy, we transfected VSMCs with si-CSNK1A1 or si-NC. In our study, we found that AKT phosphorylation levels were significantly increased by si-CSNK1A1, whereas the phosphorylation level of mTOR and total protein expression levels of AKT and mTOR did not change significantly (Fig. 7I, J). As expected, the si-CSNK1A1-induced increase in AKT phosphorylation was suppressed by MK2206 (Fig. 7K, L). We also found that LC3 (both the LC3 I and LC3 II forms) and ATG7 expression levels, which were suppressed by si-CSNK1A, were significantly increased by MK2206, whereas the LC3 II/LC3 I ratio remained unchanged (Fig. 7M, N). Collectively, these data suggest that CSNK1A1 knockdown regulates autophagy via the AKT/ATG7 pathway.

### Silencing CARMN promotes atherosclerosis by regulating autophagy in VSMCs

**Fig. 8:**
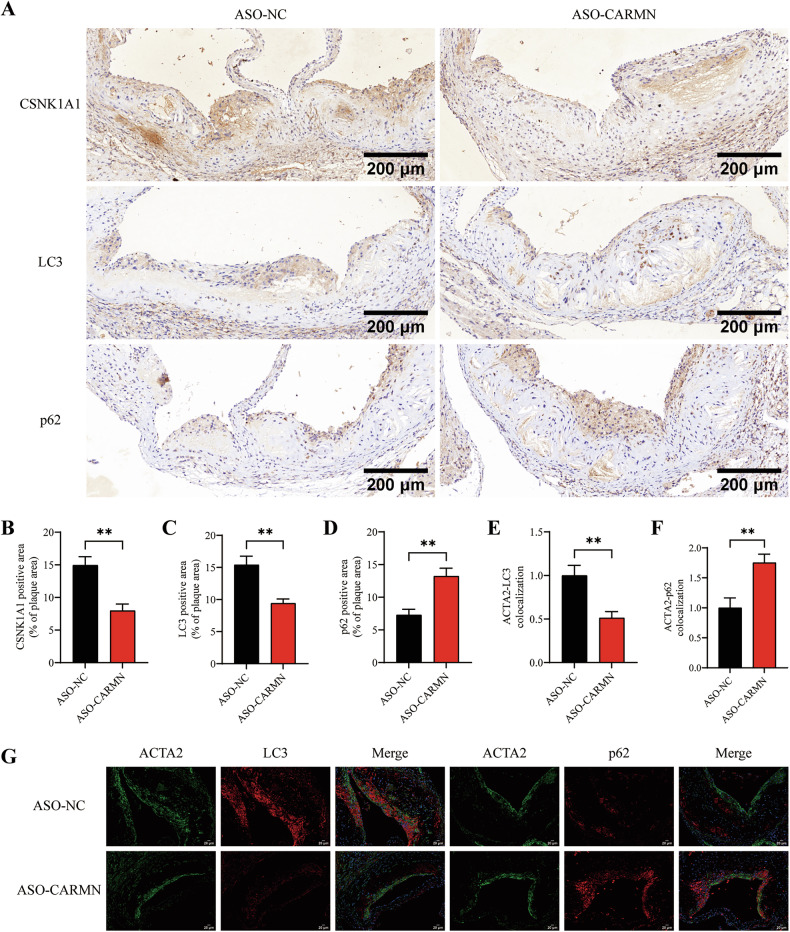
Silencing CARMN promotes atherosclerosis by regulating autophagy in VSMCs. **A** Representative images of aortic root sections stained with antibodies against CSNK1A1, LC3, or p62. Scale bars, 200 µm. **B–D** The percentages of CSNK1A1, LC3, or p62 positive area in the aortic root lesion (*n* = 6). **E**, **F** The percentages of ACTA2-LC3 colocalization and ACTA2-p62 colocalization in the plaque area (*n* = 3). **G** Representative images of immunofluorescent staining showing ACTA2-LC3 colocalization and ACTA2-p62 colocalization in aortic root sections from ASO-NC and ASO-CARMN groups. Nuclei were stained with DAPI (blue). Scale bars, 20 µm. Data are presented as mean ± SD. Statistical analysis was performed using the two-tailed Student’s t-test for two-group comparisons. **, *P* < 0.01.

We further explored whether knockdown of CARMN regulates autophagy in HFD-fed ApoE^−/−^ mice. To induce atherosclerosis, ApoE^−/−^ mice were fed a HFD for 8 weeks and then maintained on a HFD for an additional 4 weeks. During the last 4 weeks, ASO-CARMN (10 nmol per mouse) and ASO-NC (10 nmol per mouse) were administered via tail vein injection twice weekly. Using immunohistochemistry (IHC) staining, we found that LC3 expression in the ASO-CARMN-transfected group was decreased, whereas p62 expression was increased. These data suggest that CARMN knockdown regulates autophagy in ApoE^−/−^ mice. Consistent with the in vitro results, we also found that CSNK1A1 expression was decreased by ASO-CARMN (Fig. 8A–D).

To further investigate whether CARMN knockdown regulates autophagy in VSMCs in vivo, we analyzed the levels of the autophagy-related markers LC3 and p62 in the VSMCs of atherosclerotic lesions using immunofluorescence staining (Fig. 8G). We found that CARMN knockdown significantly decreased ACTA2-LC3 colocalization but increased ACTA2-p62 colocalization, suggesting that the knockdown of CARMN regulates autophagy in VSMCs in vivo (Fig. 8E, F). Collectively, our previous experimental results indicate that silencing CARMN promotes atherosclerosis; therefore, we conclude that silencing CARMN regulates autophagy in VSMCs to accelerate atherosclerotic progression in vivo.

## Discussion

Atherosclerosis is a complex chronic disease caused by multiple factors and serves as the pathologic basis of ASCVD [1]. Despite significant advances in preventive strategies and pharmacologic treatments for ASCVD, its global morbidity and mortality rates remain high [30]. Therefore, in-depth exploration of key targets in atherosclerosis and mechanistic investigations will improve our understanding of ASCVD pathogenesis and facilitate the development of more effective preventive and therapeutic approaches.

LncRNAs were previously considered nonfunctional transcriptional products. However, recent studies have demonstrated their crucial regulatory roles in gene expression through the modulation of chromatin modifications, transcription, or posttranscriptional processes [26]. Increasing evidence suggests that dysregulated lncRNA expression contributes to lipid metabolism disorder and atherosclerotic progression [31–33]. Nevertheless, many lncRNAs remain undiscovered and lack functional annotations. Our study focused on the lncRNA CARMN, which is located immediately upstream of miR-145 and miR-143. CARMN is an evolutionarily conserved lncRNA expressed in both humans and mice [27]. Silencing CARMN resulted in increased lipid deposition and necrotic core, indicating its potential as a therapeutic target for the treatment of atherosclerosis.

Various cell types, including endothelial cells, macrophages, and VSMCs, contribute to atherosclerosis progression [34–36]. Studies have confirmed that CARMN is predominantly expressed in VSMCs, and that VSMC phenotypic switching plays an important role in all stages of atherosclerosis [27]. Recent studies have demonstrated that CARMN knockdown promotes VSMC dedifferentiation by downregulating miR-145 and miR-143 expression, ultimately accelerating atherosclerosis progression [16]. However, in vitro experiments revealed that exogenous supplementation with miR-145 and miR-143 did not completely reverse the proatherogenic effects induced by CARMN silencing, suggesting that CARMN knockdown may promote atherosclerosis progression through additional mechanisms [17]. To identify the novel mechanisms through which CARMN knockdown promotes atherosclerosis, bioinformatics analysis of VSMCs revealed that the differentially expressed genes were significantly enriched in lipid metabolism following CARMN knockdown. Recent studies in human atherosclerotic plaques have shown that at least 50% of foam cells are derived from VSMCs [4, 5]. Therefore, we further investigated the effect of CARMN on VSMC-derived foam cell formation. In the present study, we demonstrated that CARMN knockdown increased VSMC-derived foam cell formation in vivo. Moreover, CARMN deficiency promoted foam cell formation by inhibiting cholesterol efflux in vitro. These findings improve our understanding of the novel mechanism by which CARMN regulates atherosclerosis.

Foam cell formation resulting from excessive cholesterol accumulation represents the defining pathological characteristic of atherosclerotic lesions. The cholesterol reverse transporters ABCA1 and ABCG1 play crucial roles in maintaining cholesterol homeostasis [4]. VSMC-derived foam cells selectively reduce ABCA1 protein expression [7]. However, we did not find that CARMN affected ABCA1 and ABCG1 expression. Studies have demonstrated that activated autophagy enhances cholesterol efflux, thereby reducing foam cell formation and alleviating the atherosclerotic burden [8, 37, 38]. In a mouse model of atherosclerosis, moderate autophagy in VSMCs promoted cholesterol efflux and reduced intracellular lipid accumulation, thereby inhibiting lipid deposition and necrotic core formation in atherosclerotic lesions [39]. In advanced atherosclerosis, the P2RY12 receptor decreases cholesterol efflux and promotes VSMC-derived foam cell formation by inhibiting autophagy [13]. A recent study suggested that atherosclerosis impairs the autophagic function of both macrophage-derived and VSMC-derived foam cells, with the latter being more severely affected. The autophagy activator metformin enhances cholesterol efflux and inhibits foam cell formation by activating autophagy in VSMCs [40]. These studies suggest that autophagy is closely related to VSMC-derived foam cell formation. Therefore, we hypothesized that CARMN regulates the formation of VSMC-derived foam cells through autophagy. In the present study, we found that the knockdown of CARMN transcriptionally downregulates autophagy in VSMCs. Furthermore, CARMN knockdown reduces cholesterol efflux and promotes VSMC-derived foam cell formation by regulating autophagy. These findings improve our understanding of the mechanisms underlying VSMC-derived foam cell formation and suggest that enhancing VSMC autophagy is a potential treatment for atherosclerosis.

To identify the exact mechanism by which CARMN regulates autophagy in VSMCs, transcriptomic analysis of VSMCs was performed, which revealed that knockdown of CARMN significantly changed the expression levels of HIF1α, Raptor, DAPK, ATG9L, ATG10, and FLIP. These genes are involved in the nucleation process of autophagic vacuoles, suggesting that autophagy is regulated in the initial phase and is involved in two possible mechanisms: the initial step of vesicle nucleation or signaling pathways upstream of autophagy, such as the AMPK or AKT-mTOR pathways. Increasing evidence suggests that the AKT-mTOR signaling pathway plays a key role in autophagy [24, 25]. However, CARMN knockdown altered AKT phosphorylation but not mTOR phosphorylation, suggesting that CARMN does not regulate autophagy through the classical AKT-mTOR signaling pathway. ATG7 is a crucial initiator of the autophagy pathway and functions as an E1-like enzyme that activates both ATG12 and ATG8. This activation facilitates the subsequent conjugation of ATG12 to ATG5 and ATG8 to phosphatidylethanolamine, thus initiating autophagosome formation [41]. Research findings indicate that targeted knockdown of ATG7 in VSMCs accelerates plaque formation in ApoE^−/−^ mice [42]. Furthermore, a recent study revealed that ATG7 silencing promotes the formation of VSMC-derived foam cells by inhibiting autophagy [43]. To determine whether CARMN regulates autophagy through the AKT/ATG7 pathway, we used MK2206 (an AKT inhibitor) and found that CARMN knockdown regulates autophagy via this pathway. Thus, inhibiting the AKT/ATG7 pathway in VSMCs may be an effective strategy for treating atherosclerosis.

In most cases, lncRNAs exert their functions by regulating the expression of target mRNAs at the transcriptional or posttranscriptional level [26]. To further elucidate the potential mechanism of CARMN, we identified CSNK1A1 as a direct binding protein of CARMN through online prediction using the RPISeq database and the CatRAPID algorithm, followed by validation with RIP experiments. We found that CARMN knockdown significantly suppressed CSNK1A1 expression, suggesting that CARMN knockdown inhibits the transcription of the adjacent gene CSNK1A1. A recent study revealed that CSNK1A1 inhibits autoimmune diseases by promoting the autophagic degradation of STING1 [44]. In this study, we found that the knockdown of CSNK1A1 transcriptionally downregulates autophagy in VSMCs. A previous study revealed that silencing CSNK1A1 regulates autophagy through the activation of AKT phosphorylation, with no effect on mTOR activity [45]. Another study showed that CSNK1A1 inhibits the growth of non-small cell lung cancer by inducing autophagy via the AKT/FOXO3a/ATG7 pathway without affecting mTOR activity [29]. Consistent with these two previous studies, we found that CSNK1A1 knockdown altered AKT phosphorylation but not mTOR phosphorylation. Furthermore, we investigated the potential mechanism underlying how CSNK1A1 regulates autophagy and reported that knockdown of CSNK1A1 regulates autophagy via the AKT/ATG7 pathway. These findings demonstrate that the CARMN-CSNK1A1 pathway is essential for regulating autophagy. By investigating the mechanism by which CARMN regulates atherosclerosis, we found that silencing CARMN regulates autophagy in VSMCs, thereby accelerating atherosclerotic progression in vivo.

These data reveal the role of lncRNAs in regulating atherosclerosis. We identified CARMN as a novel regulator of autophagy and elucidated the molecular mechanism underlying its regulatory effect on CSNK1A1 in VSMCs. However, our study has several limitations that should be acknowledged. First, the lack of lipid uptake assays in our in vitro experiments may affect the interpretation of our findings. Future studies incorporating lipid uptake assays could better characterize the impact of CARMN on VSMC-derived foam cell formation. Second, although we demonstrated that knockdown of CARMN resulted in significant downregulation of CSNK1A1 in vivo, we did not directly validate the biological role of CSNK1A1 in atherosclerosis. Further studies are warranted to clarify the in vivo function of CSNK1A1 in the progression of atherosclerosis. Furthermore, the clinical significance of our findings remains unexplored, and further studies are needed to investigate the therapeutic potential of CARMN homologous transcripts in autophagy-related diseases.

**Fig. 9:**
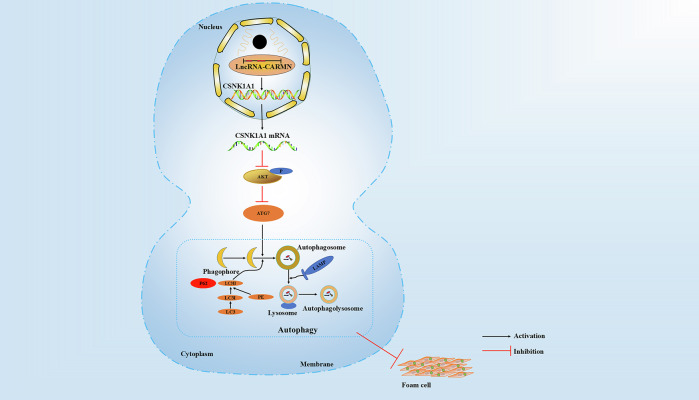
Schematic diagram of the main findings of this study. Our study demonstrated that CARMN knockdown suppressed CSNK1A1 expression, thereby promoting VSMC-derived foam cell formation and atherosclerosis via the transcriptional downregulation of autophagy mediated by the AKT/ATG7 pathway. (Black arrows represent activation, and red arrows represent inhibition).

In summary, as shown in the schematic diagram (Fig. 9), our study demonstrated that CARMN knockdown suppressed CSNK1A1 expression, thereby promoting VSMC-derived foam cell formation and atherosclerosis via the transcriptional downregulation of autophagy mediated by the AKT/ATG7 pathway. Our results shed light on how the links between CARMN and lipophagy affect VSMC-derived foam cell formation. CSNK1A1 expression regulated by CARMN in the AKT/ATG7 pathway reveals a novel mechanism for the regulation of autophagy in VSMCs. Our research elucidates the role and mechanism of CARMN in atherosclerosis progression, suggesting potential therapeutic approaches to combat atherosclerotic plaque progression.

## Material and methods

### Data acquisition and processing

We acquired 222 ATGs from the human autophagy database (http://www.autophagy.lu/index.html). The lncRNA expression profile GSE97210 and the mRNA expression profile GSE158972 were downloaded from the Gene Expression Omnibus (GEO) database (http://www.ncbi.nlm.nih.gov/geo/). The GSE97210 dataset is based on the GPL16956 platform (Agilent-045997 Arraystar human lncRNA microarray V3) and consists of 3 human normal arterial intimal tissues and 3 human atherosclerotic plaque tissues. The GSE158972 dataset is based on the GPL20301 platform (Illumina HiSeq 4000) and consists of 18 control human-derived VSMCs and 18 human-derived VSMCs with knockdown of the lncRNA CARMN. Sample collection and processing for GSE97210 were performed in accordance with the guidelines of the Committee for Ethical Review of Research Involving Human Subjects, Nanfang Hospital, Southern Medical University, Guangzhou, China (approval no. NFEC-2018-142), and oral informed consent was obtained from the participants or their relatives. The probe IDs in the expression matrix were processed into gene symbols using the annotation file in the gene chip.

### Identification of DEGs

PCA was performed using the “FactoMineR” and “factoextra” packages. The DEGs were identified using the “limma” package. The heatmaps and volcano plots were generated with the “heatmap” and “ggplot2” packages.

### Functional enrichment analysis

GO and KEGG enrichment analyses of the DEGs were performed using the “clusterProfiler” and “Goplot” packages in R software (version 4.3.0). The results were visualized with bubble plots and chord diagrams.

### Reagents and antibodies

The following reagents were used: Bodipy 493/503 (Amgicam, ajci70160; 5 µM), NBD-cholesterol (Biofount, HCQ000646; 10 μM), CQ (Selleck Chemicals, S6999; 50 μM), MK2206 (Selleck Chemicals, S1078; 5 µM), Rapa (Selleck Chemicals, S1039; 100 nM), ox-LDL (Yiyuan Biotech, YB-002; 50 μg/mL) and HDL (Yiyuan Biotech, YB-003; 50 μg/mL). The following primary antibodies were used for western blot experiments: anti-ABCA1 (ABclonal, A16337), anti-ABCG1 (Proteintech, 13578-1-AP), anti-ATG7 (Proteintech, 10088-2-AP), anti-p62 (Proteintech, 18420-1-AP), anti-LC3 (ABclonal, A19665), anti-CSNK1A1 (ABclonal, A9308), anti-AKT (Cell Signaling Technology, 4691 T), anti-P-AKT-T308 (Cell Signaling Technology, 13038 T), anti-mTOR (Proteintech, 66888-1-Ig), anti-P-mTOR-S2448 (Cell Signaling Technology, 5536S), anti-β-actin (Proteintech, 20536-1-AP), and anti-GAPDH (Proteintech, 60004-1-Ig). The following primary antibodies were used for IHC staining: anti-CSNK1A1 (Proteintech, 55192-1-AP), anti-LC3 (Proteintech, 14600-1-AP), and anti-p62 (Proteintech, 18420-1-AP). The following primary antibodies were used for immunofluorescence staining: anti-LC3 (Proteintech, 14600-1-AP), anti-p62 (Proteintech, 18420-1-AP), and anti-ACTA2 (Proteintech, 67735-1-Ig). The following primary antibodies were used for RIP assay: anti-CSNK1A1 (Proteintech, 55192-1-AP) and anti-IgG (Proteintech, 25851-1-AP). The following secondary antibodies were used for western blot experiments: anti-rabbit (Abbkine, A21020) and anti-mouse (Abbkine, A21010) HRP-conjugated IgGs. The following secondary antibodies were used for IHC staining: anti-rabbit (Proteintech, SA00001-2) HRP-conjugated IgGs. The following secondary antibodies were used for immunofluorescence staining: anti-rabbit Alexa Fluor 594-conjugated (Thermo Fisher Scientific, A11012), anti-mouse Alexa Fluor 594-conjugated (Thermo Fisher Scientific, R37115), and anti-mouse Alexa Fluor 488-conjugated (Thermo Fisher Scientific, A11011).

### Atherosclerotic mouse models

Male C57BL/6 J mice and ApoE^−/−^ (C57BL/6 J background) mice, 8 weeks of age (21–25 g), were purchased from the Vital River Laboratory Animal Center (Beijing, China) and housed under a 12 h light/dark cycle with free access to drinking water and food in an environmentally controlled room (20–26 °C, 60% humidity). C57BL/6 J mice were fed a normal chow diet containing 4% fat and 0% cholesterol for 8 weeks and were used as the normal control group. Eight-week-old ApoE^−/−^ mice were fed a HFD containing 20% fat and 1.25% cholesterol (Medicience Ltd., D12108C) for 8 weeks to induce atherosclerotic plaque formation.

The mice that formed atherosclerotic plaques were then randomly divided into two groups, the ASO-NC group and the ASO-CAMN group, and the HFD was continued for 4 weeks. During the last 4 weeks, ASO-NC (10 nmol per mouse) and ASO-CARMN (10 nmol per mouse) were administered via tail vein injection twice weekly. The mice were sacrificed with carbon dioxide (CO_2_) in accordance with the NIH Guidelines and then used for aortic tissue collection and follow-up examinations. The negative control (ASO-NC) and ASO-CARMN were purchased from RiboBio (Guangzhou, China).

### Atherosclerotic lesion analysis

The entire aorta from the ascending aorta to the bifurcation of the iliac arteries was isolated, stripped of peripheral vascular fat, and incised longitudinally. The samples were subsequently stained with ORO dye (Servicebio, G1015) and photographed with a digital camera. The percentage of area occupied by the ORO-positive area was assessed using the ImageJ software.

### Histology and immunofluorescence staining

The heart and aortic tissues were fixed in 4% PFA for 24 hours. The aortic root tissues were both paraffin-embedded and frozen-embedded, with the thickness of the paraffin section being 5 µm and the frozen section being 8 µm. HE, Masson, and IHC staining were performed on paraffin sections of aortic root tissues. For immunofluorescence staining, frozen sections were incubated with antibodies against LC3 (Proteintech, 14600-1-AP), p62 (Proteintech, 18420-1-AP), or ACTA2 (Proteintech, 67735-1-Ig). VSMCs were fixed, permeabilized, and then incubated with an antibody against LC3 (Proteintech, 14600-1-AP). The immune complexes were detected with fluorescently labeled secondary antibodies, and 4’,6-diamidino-2-phenylindole (DAPI) (Servicebio, G1012) was used to stain the nuclei. The stained samples were imaged by an Olympus IX73 fluorescence microscope (Olympus, Tokyo, Japan) or a Zeiss LSM 710 confocal microscope (Carl Zeiss, Oberkochen, Germany). Images were quantified using the ImageJ software. Specifically, for histology images, we first used the “Split Channels” function in ImageJ to obtain the grayscale image of the target channel. After selecting an appropriate threshold to define the positive area, we applied the “Measure” function to quantify the area. For single-antibody immunofluorescence and BODIPY colocalization images, we first used the “Split Channels” function in ImageJ to obtain grayscale images of the target channels. The JACop plugin was then used to assign the channels as Image A and Image B. After appropriate thresholds were selected to determine the colocalized area, the “Analyze” function was used to obtain Manders’ coefficient. Quantification of the colocalized area was performed on the basis of Manders’ coefficient. For LC3 immunofluorescence images, we first used the “Split Channels” function in ImageJ to obtain grayscale images of the target channel. After applying an appropriate threshold, we used the “Analyze Particles” function to quantify the number of LC3 dots.

### Cell culture

Mouse aortic VSMCs (AW-CNM518) were purchased from Abiowell Biotechnology Co., Ltd. (Changsha, China). The cells were grown in Dulbecco’s modified Eagle’s medium (Gibco, 105690010) supplemented with 10% fetal bovine serum at 37 °C and 5% CO_2_ in a humidified incubator. The cells were seeded in 6- or 12-well plates or 60-mm dishes and grown to 70-80% confluence before use.

### siRNA and ASO transfection

CSNK1A1 siRNA and ATG7 siRNA were synthesized by RiboBio (Guangzhou, China). The siRNAs were transfected into cells using Lipofectamine 3000 (Invitrogen, USA) according to the manufacturer’s protocol. The cells were cultured for 48 h for subsequent experiments. The CARMN ASO was purchased from RiboBio (Guangzhou, China). The ASO transfection process was similar to that of the siRNAs; an ASO transfection kit (RiboBio) was used, with a 48 h incubation period.

### Plasmid transfection

The plasmids overexpressing CARMN or CSNK1A1 were purchased from Igebio (Guangzhou, China). For plasmid transfection, exponentially growing cells were seeded in six-well plates and then transfected with the plasmid CARMN or CSNK1A1 vector using Lipofectamine 3000. The transfected cells were cultured for 48 h for subsequent experiments.

### BODIPY staining

LDs were stained by incubating the cells or sections with BODIPY 493/503 for 30 min and then with DAPI for 10 min. The stained samples were imaged by an Olympus IX73 fluorescence microscope (Olympus, Tokyo, Japan) or a Zeiss LSM 710 confocal microscope (Carl Zeiss, Oberkochen, Germany). Quantification was performed using ImageJ software. Specifically, for BODIPY staining images, we first used the “Split Channels” function in ImageJ to obtain grayscale images of the target channel. After applying an appropriate threshold, the “Analyze Particles” function was employed to count the number of LDs, followed by the “Measure” function to quantify the area of LDs.

### Cholesterol efflux assay

VSMCs were seeded in 6-well plates and incubated with NBD-cholesterol for 4 h at 37 °C. After cholesterol loading, the cells were washed twice with PBS, the medium was replaced with phenol red-free DMEM to equilibrate the cells for 12 h, and the cells were then incubated with HDL for 4 h. The supernatant was collected, and phenol red-free DMEM was added to the cells, which were then collected in a centrifuge tube. The cells were lysed by sonication for 10 min (frequency of 25 kHz; sonication for 2 s with 2 s intervals) until the cells were completely lysed and the solution was clarified. The collected supernatant and cell lysate were added to a 96-well lightproof plate, and the fluorescence value was detected by a multifunctional enzyme labeling instrument with an emission wavelength of 535 nm and an excitation wavelength of 485 nm. The efflux rate was calculated as follows: Efflux (%) = [supernatant value/(supernatant value + cell lysate value)] × 100.

### Autophagic flux

VSMCs in the logarithmic growth phase were seeded in 12-well plates and transfected with ad-mRFP-GFP-LC3 (HanBio Technology, Shanghai, China) when the cell confluence reached 30-40%. Twenty-four hours after transfection, the cells were washed with sterile PBS, treated with the corresponding treatments, and cultured for 24 h. Then, the cells were fixed with 4% PFA, and the nuclei were stained with DAPI. The cells were observed with an Olympus IX73 fluorescence microscope (Olympus, Tokyo, Japan) to detect autophagic flux. The numbers of mRFP^+^-GFP^+^ and mRFP^+^-GFP^−^ puncta were analyzed using ImageJ software.

### Electron microscopy

The cells were inoculated in Petri dishes and fixed with 2.5% glutaraldehyde at the end of the corresponding stimulation. After being embedded, the cells were cut into 60-80 nm thin slices with an ultrathin sectioning machine and finally double-stained with uranium lead. The cells were observed under a transmission electron microscope (Tecnal G^2^ 20 TWIN, FEI, USA), and images were captured for further analysis.

### RIP assay

For the RIP assay, the cells were harvested, washed with ice-cold PBS buffer, lysed in RIP buffer consisting of IP lysis buffer (Beyotime, P0013) and an RNase inhibitor (Beyotime, R0102), and then centrifuged for 15 min at 12,000 rpm. The cell lysates were incubated with the indicated antibodies at 4 °C overnight. Approximately 40 μL of protein A/G magnetic beads were added to the reaction mixtures, which were subsequently incubated for 4 h at 4 °C. After magnetic separation, the magnetic beads were washed five times with RIP buffer. Finally, the purified RNA was subjected to qRT-PCR analysis. The antibodies used for RIP included CSNK1A1 (Proteintech, 55192-1-AP) and IgG (Proteintech, 25851-1-AP).

### Western blotting

The cells were lysed in RIPA buffer (Coolaber, SL1020), and the protein lysates were quantified. Equal amounts of protein lysates were subjected to SDS‒PAGE. The separated proteins were transferred onto PVDF membranes. The membranes were blocked with 5% skim milk for 1 h at room temperature. The membranes were subsequently incubated overnight at 4 °C with primary antibodies against the following proteins: ABCA1, ABCG1, ATG7, p62, LC3, CSNK1A1, AKT, P-AKT-T308, mTOR, and P-mTOR-S2448. β-actin or GAPDH was used as a loading control. The membranes were subsequently incubated with the corresponding secondary antibodies. The protein band signals were developed by an ECL detection system (WBKLS0500, Merck Millipore).

### qRT-PCR

Total RNA was extracted from VSMCs and aortas using TRIzol reagent (Invitrogen, 15596026). cDNA was subsequently generated from the total RNA using the RevertAid First Strand cDNA Synthesis Kit (Thermo Fisher, K1622). Real-time qPCR was performed using SYBR Green Master Mix (Yeasen Biotechnology, 11201ES08). The individual mRNA expression levels were normalized to those of GAPDH. The primers for CARMN were 5′-GGAGAAGAGCCCCAGAGAGG-3′ (forward) and 5′-TTCTGTCCGTTGGGAAGCTC-3′ (reverse). The primers for CSNK1A1 were 5′-ACAACAGGACAAGGCAACACATACC-3′ (forward) and 5′-CAACACCTCAACAGGAGTGGACATC-3′ (reverse). The primers for GAPDH were 5′-TGTTTCCTCGTCCCGTAG-3′ (forward) and 5′-CAATCTCCACTTTGCCACT-3′ (reverse).

### Statistical analysis

All data are presented as the mean ± SD, and the normality of the data was determined using the Shapiro–Wilk normality test. Two-tailed Student’s *t* test was used for two-group comparisons, and one-way ANOVA was employed for multiple-group comparisons. Statistical analysis was performed using the GraphPad Prism 9 software and R software. *P* < 0.05 was considered statistically significant.

## Supplementary information

Original western blots

Supplemental Figure

## Data Availability

The datasets GSE97210 and GSE158972 for this study can be freely and openly accessed in the GEO database (https://www.ncbi.nlm.nih.gov/geo/). The codes used in the manuscript are available at https://github.com/HuangTG0120/atherosclerosis. All other data are available from the corresponding author upon reasonable request.
