# Supplementary figures and images for: CARMN loss promotes VSMC-derived foam cell formation and atherosclerosis through transcriptional downregulation of autophagy
Source: Cell Death Dis. 2025 Nov 10;16(1):815. doi: 10.1038/s41419-025-08157-z (PMC12603328; doi:10.1038/s41419-025-08157-z)

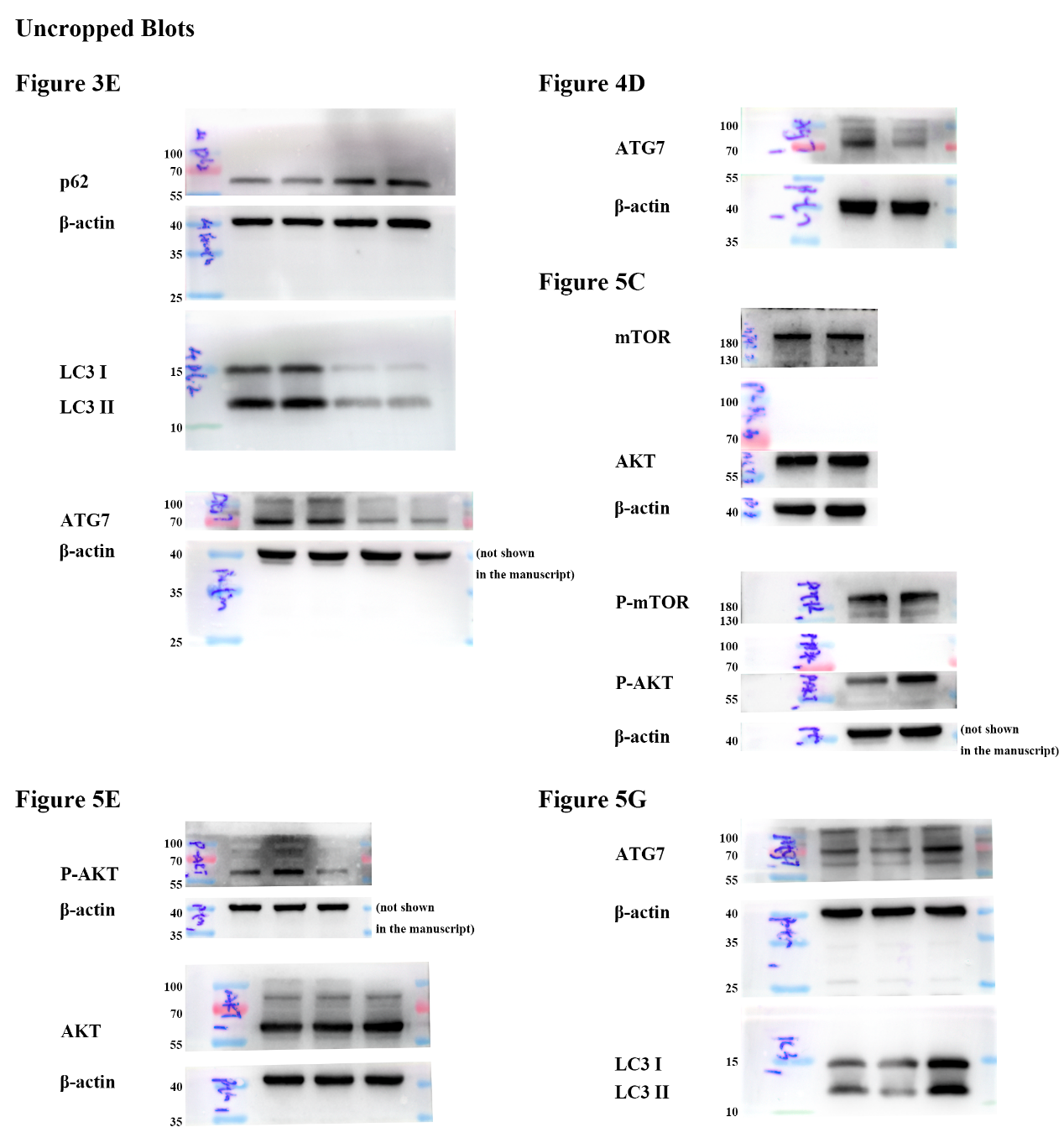


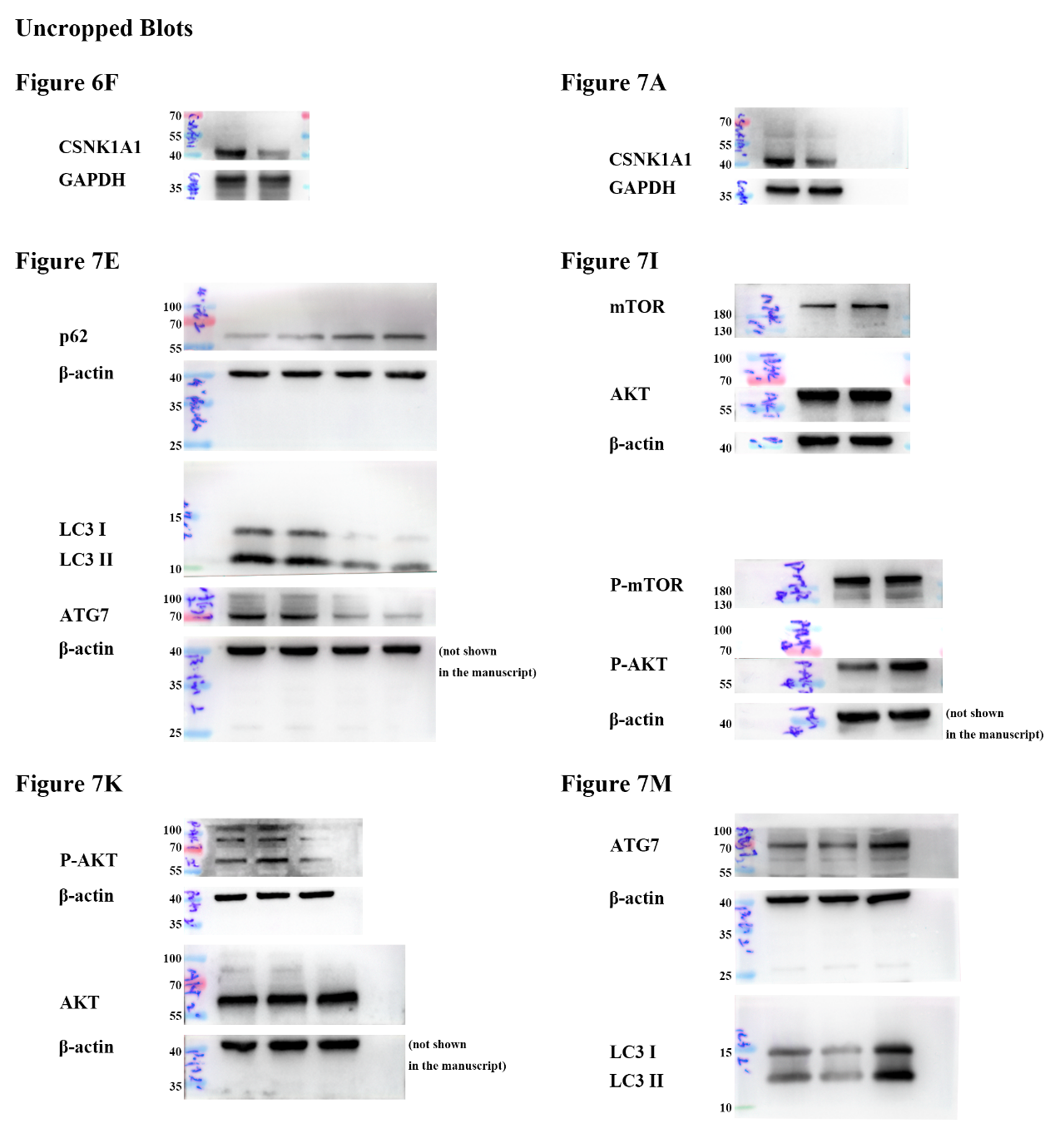


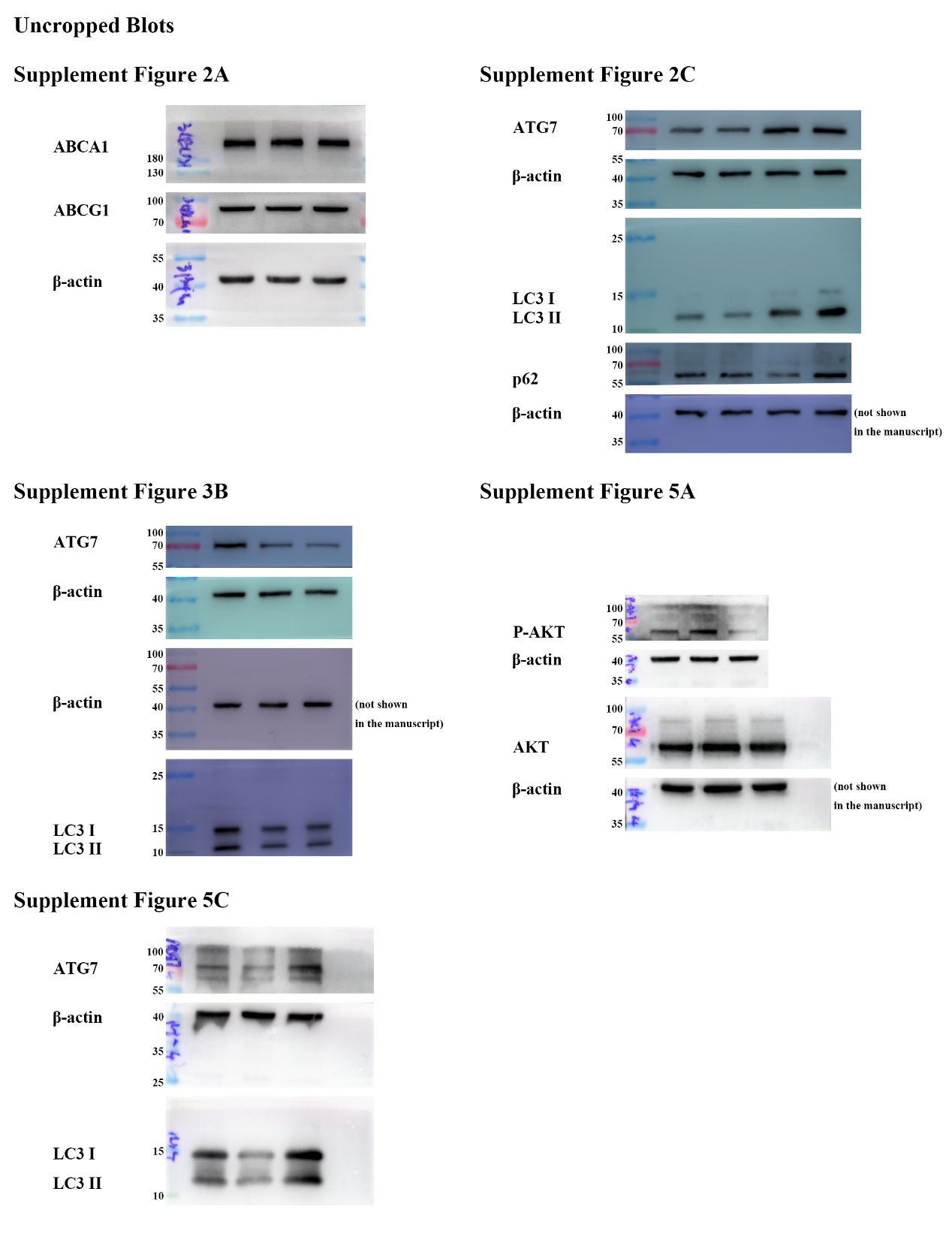

Supplement: Supplementary file 1 — Original western blots [file 41419_2025_8157_MOESM1_ESM.docx]
