# Supplementary material for: CARMN loss promotes VSMC-derived foam cell formation and atherosclerosis through transcriptional downregulation of autophagy
Source: Cell Death Dis. 2025 Nov 10;16(1):815. doi: 10.1038/s41419-025-08157-z (PMC12603328; doi:10.1038/s41419-025-08157-z)
Supplement: Supplementary file 2 — Supplemental Figure [file 41419_2025_8157_MOESM2_ESM.pdf]

SUPPLEMENTAL MATERIAL

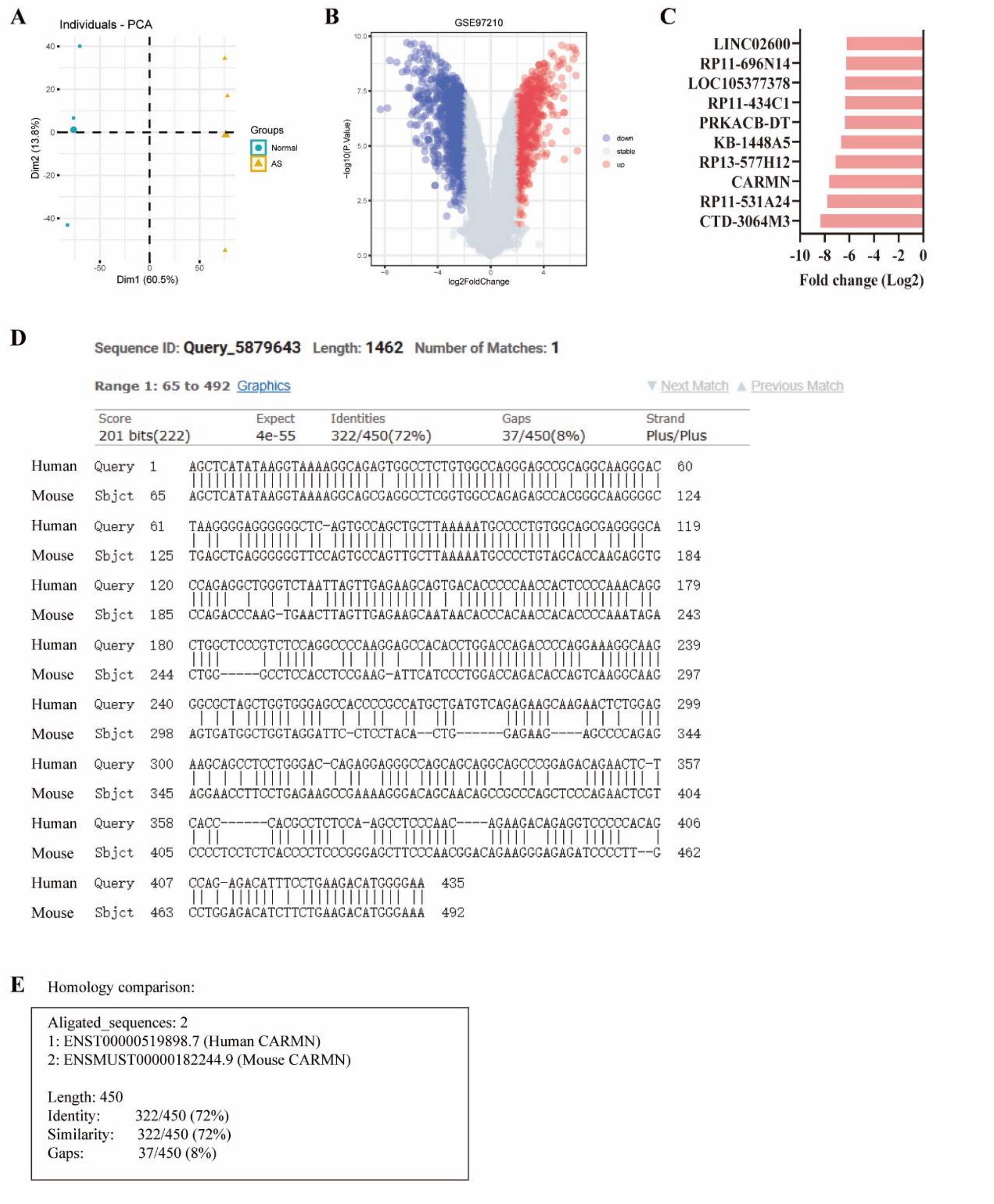

Figure S1.

(A) The PCA plot of GSE97210 of the normal arterial intimal tissues and atherosclerotic plaque tissues in humans. **(B)** Volcano plot showing the differentially expressed lncRNAs in the normal arterial intimal tissues and atherosclerotic plaque tissues. Red and blue dots represent significantly upregulated and downregulated genes with $|log_2FC| > 2$ and $P < 0.05$ as the threshold parameters. The gray dots indicate non-differentially expressed genes. **(C)** The fold change in expression of the top ten downregulated lncRNAs in the atherosclerosis group compared to the normal group. **(D)** CARMN homology analysis in the human and mouse genomes was conducted using BLAST. **(E)** Homology comparison of human and mouse CARMN.

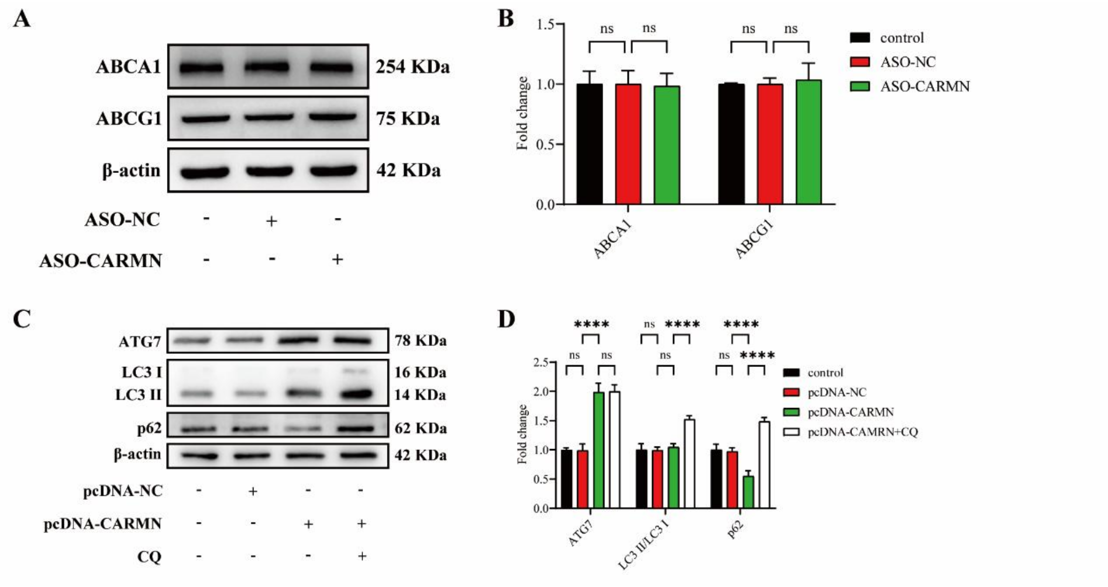

Figure S2.

(A) Western blotting analysis of the expression of ABCA1 and ABCG1 in VSMCs treated with vehicle control, ASO-NC, or ASO-CARMN. **(B)** Quantification of ABCA1 and ABCG1 protein expression (n=3). **(C)** Western blotting analysis of the expression of LC3, p62, and ATG7 in VSMCs incubated with CQ (50 μM) for 12 h before treatment with pcDNA-NC or pcDNA-CARMN. (**D)** Quantification of LC3 II/LC3 I, p62 and ATG7 protein expression (n=3). Data are presented as mean ± SD. Statistical analysis was performed using one-way ANOVA for multiple-group comparisons. ns, $P > 0.05$; ****, $P < 0.0001$.

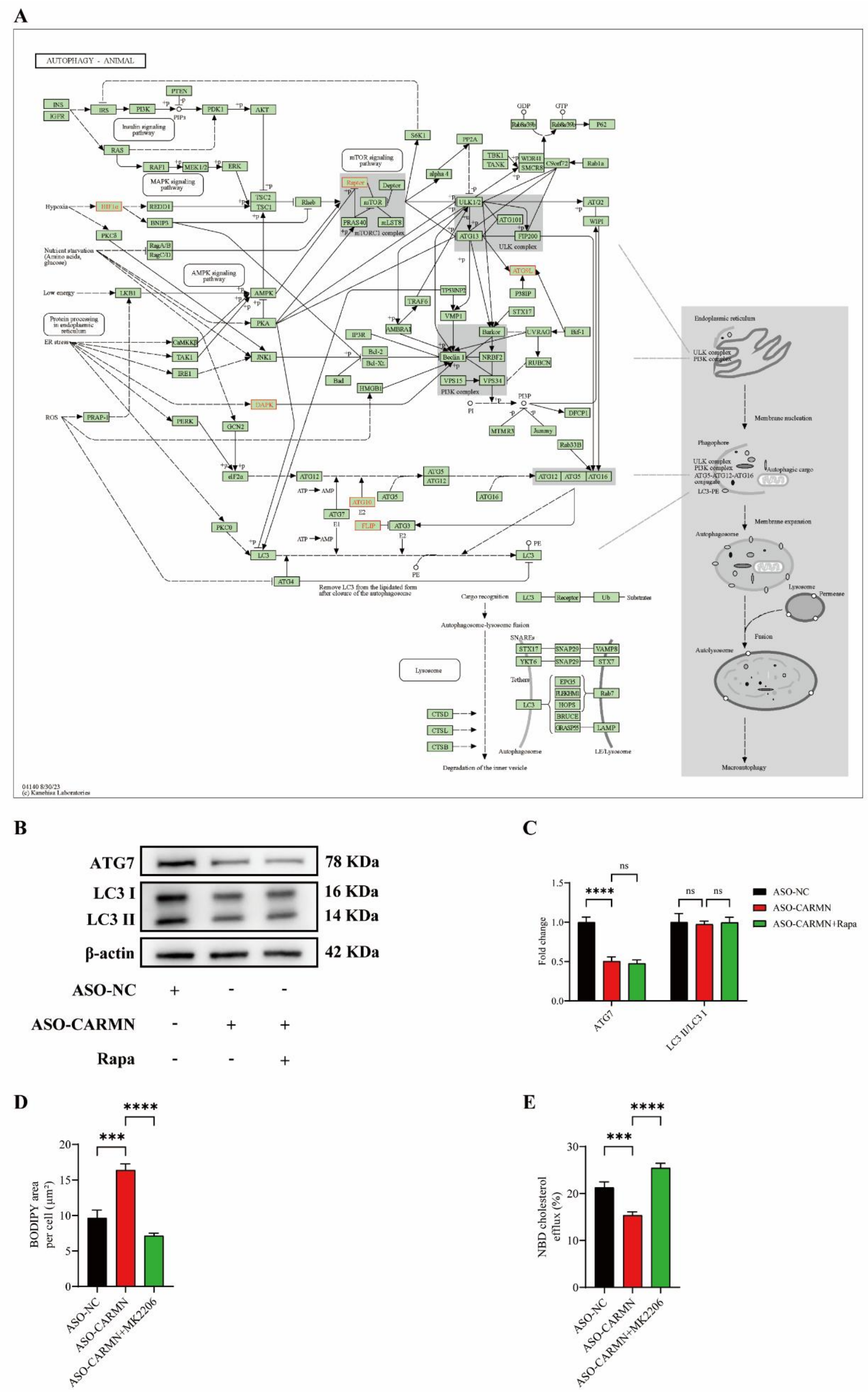
A
AUTOPHAGY - ANIMAL
Insulin signaling pathway
MAPK signaling pathway
mTOR signaling pathway
AMPK signaling pathway
mTORC1 complex
ULK complex
PI3K complex
Hypoxia
Nutrient starvation (Amino acids, glucose)
Low energy
Protein processing in endoplasmic reticulum
ER stress
ROS
Remove LC3 from the lipidated form after closure of the autophagosome
Cargo recognition
Autophagosome-lysosome fusion
SNAREs
Tethers
Lysosome
Autophagosome
LE/Lysosome
Degradation of the inner vesicle
Endoplasmic reticulum
Membrane nucleation
Phagophore
Autophagic cargo
Membrane expansion
Autophagosome
Lysosome
Permease
Fusion
Autolysosome
Macroautophagy
04140 8/30/23
(c) Kanehisa Laboratories
B
ATG7
78 KDa
LC3 I
16 KDa
LC3 II
14 KDa
β-actin
42 KDa
ASO-NC
ASO-CARMN
Rapa
C
Fold change
ATG7
LC3 II/LC3 I
ASO-NC
ASO-CARMN
ASO-CARMN+Rapa
ns
D
BODIPY area per cell (μm²)
ASO-NC
ASO-CARMN
ASO-CARMN+MK2206
E
NBD cholesterol efflux (%)
ASO-NC
ASO-CARMN
ASO-CARMN+MK2206

Figure S3.

(A) The "autophagy-animal" pathway obtained from the KEGG enrichment analysis. **(B)** Western blotting analysis of the expression of LC3 and ATG7 in VSMCs incubated with RAPA (100 nM) for 12 h before treatment with ASO-NC or ASO-CARMN. (**C)** Quantification of LC3 II/LC3 I and ATG7 protein expression (n=3). **(D)** The average BODIPY-positive area per cell was quantified (n=3). **(E)** HDL-mediated cholesterol efflux (%) was measured in indicated groups (n=3). Data are presented as mean ± SD. Statistical analysis was performed using one-way ANOVA for multiple-group comparisons. ns, $P > 0.05$; ***, $P < 0.001$; ****, $P < 0.0001$.

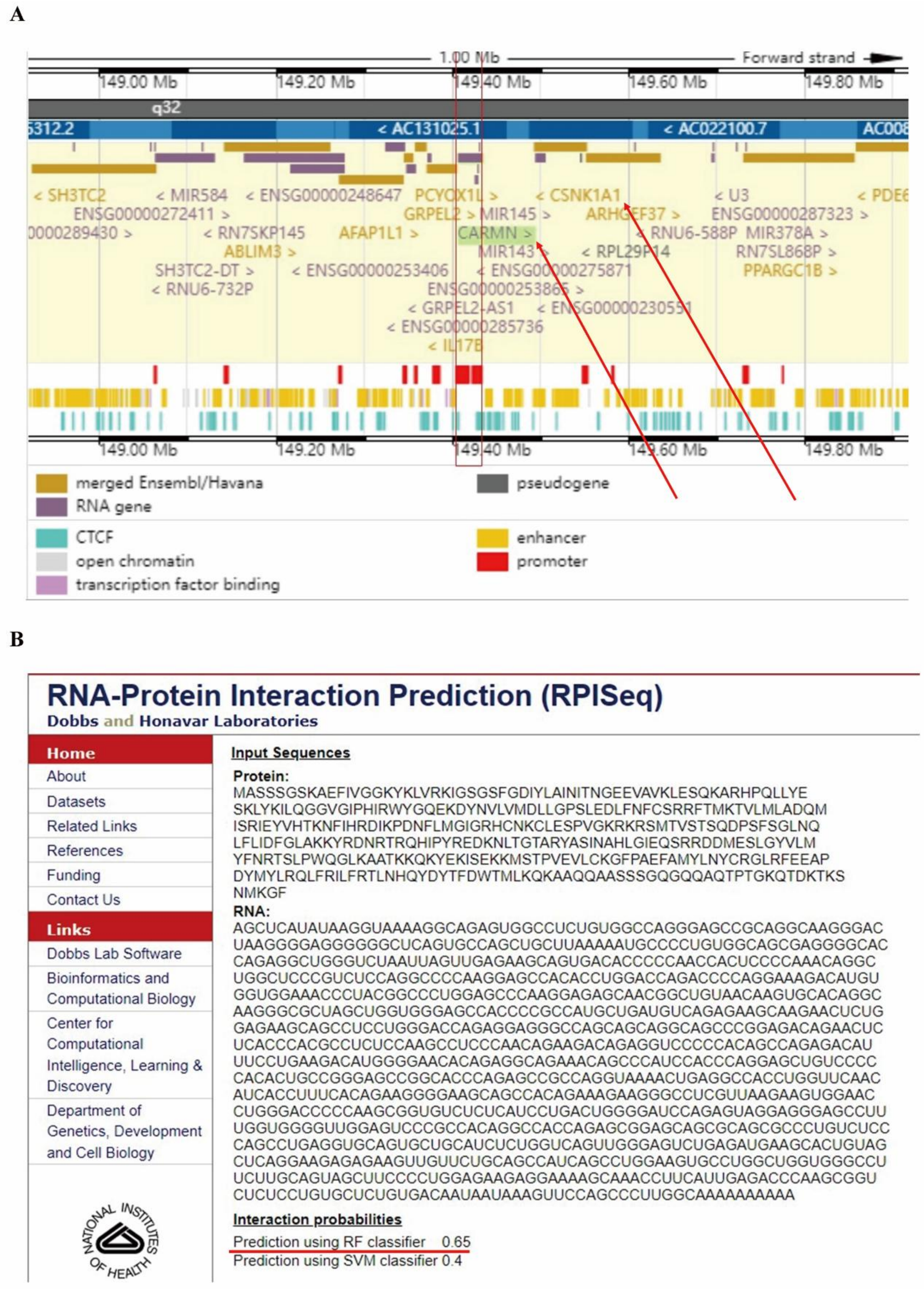

Figure S4.

(A) Adjacent genes within 100 kb upstream and downstream of CARMN in the Ensembl database. **(B)** Interaction probabilities between CARMN and adjacent gene CSNK1A1.

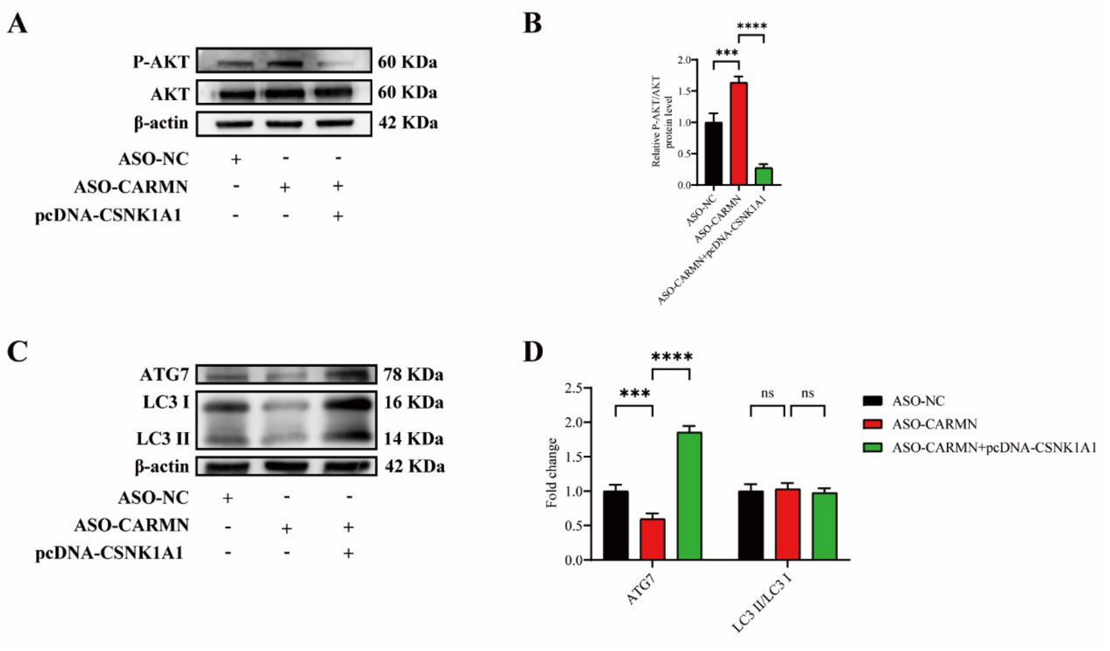

Figure S5.

(A) Western blotting analysis of total and phosphorylated forms of AKT in VSMCs treated with ASO-NC, ASO-CARMN, or ASO-CARMN plus pcDNA-CSNK1A1. **(B)** Quantification of total and phosphorylated levels of AKT (n=3). **(C)** Western blotting analysis of LC3 and ATG7 in VSMCs treated with ASO-NC, ASO-CARMN, or ASO-CARMN plus pcDNA-CSNK1A1. **(D)** Quantification of LC3 II/LC3 I and ATG7 protein expression (n=3). Data are presented as mean ± SD. Statistical analysis was performed using one-way ANOVA for multiple-group comparisons. ns, $P > 0.05$; ***, $P < 0.001$; ****, $P < 0.0001$.
